# Alternative Splicing in Next Generation Sequencing Data of *Saccharomyces cerevisiae*

**DOI:** 10.1371/journal.pone.0140487

**Published:** 2015-10-15

**Authors:** Konrad Schreiber, Gergely Csaba, Martin Haslbeck, Ralf Zimmer

**Affiliations:** 1 Institut für Informatik, Ludwig-Maximilians-Universität München, München, Germany; 2 Department Chemie, Technische Universität München, Garching, Germany; International Centre for Genetic Engineering and Biotechnology, ITALY

## Abstract

mRNA splicing is required in about 4% of protein coding genes in Saccharomyces cerevisiae. The gene structure of those genes is simple, generally comprising two exons and one intron. In order to characterize the impact of alternative splicing on the S. cerevisiae transcriptome, we perform a systematic analysis of mRNA sequencing data. We find evidence of a pervasive use of alternative splice sites and detect several novel introns both within and outside protein coding regions. We also find a predominance of alternative splicing on the 3’ side of introns, a finding which is consistent with existing knowledge on conservation of exon-intron boundaries in S. cerevisiae. Some of the alternatively spliced transcripts allow for a translation into different protein products.

## Introduction

Genes containing introns can produce several transcripts and protein products via alternative use of exons and introns. Alternative splicing (AS) exploits these gene structures to increase transcriptome and proteome complexity and to serve regulatory purposes in higher eukaryotes.

Splicing requires a complex molecular machinery, the spliceosome, which is directed by three important splice signals: the 5’ splice site, the branch point and the 3’ splice site with associated polypyrimidine tract. These signals are detected by components of the spliceosome and define the exon-intron boundaries. To enable AS in higher eukaryotes, many auxiliary factors are present such as SR proteins or hnRNPs [1]. These can enhance or block the recognition of splicing signals and play an important role in the regulation of AS.

In S. cerevisiae, about 4% of all genes contain introns and the organism is able to correctly splice those genes. While complete splicing is well studied in S. cerevisiae, the use of alternative splice sites is only described in individual examples, which we will review below. These examples have been reported in the literature and are not annotated in resources like the Saccharomyces genome database (SGD) or Ensembl. The simple gene structure and certain properties of the S. cerevisiae genome suggest that AS in S. cerevisiae is limited: its genome shows strong 5’ splice sites and a highly conserved branch point sequence (TACTAAC), which facilitate the splicing of introns [2]. Furthermore, only few auxiliary splicing factors exist [3]. However, recently several studies reported the use of alternative splicing in S. cerevisiae [4, 5].

Individual examples of AS have been described, especially in the context of environmental stress or certain states of the cell cycle. Intron retention and subsequent nonsense mediated decay (NMD) are reported for certain genes during mitosis [6] or by autoregulation under high transcript levels [7, 8]. Intron retention has also been observed to be allele specific [9]. Another mechanism, spliceosome mediated decay (SMD), targets genes lacking conventional introns and regulates their transcript levels [5]. A whole class of genes for ribosomal proteins, are regulated by enhanced or impaired splicing under normal conditions compared to amino acid starvation [10]. The gene MATa1 in the mating locus of S. cerevisiae has been observed in four isoforms, three of which are reported inactive. Those appear to be removed by nuclear RNA turnover instead of cytosolic NMD [11].

More complex AS has been confirmed on the transcript level, leading to multiple alternative mRNAs for a single gene locus [12]. Intron retention and even exon skipping have been reported and splicing is required for cellular function in the gene SUS1 [13]. Alternative isoforms of certain genes involved in gene fusion events could be confirmed on the cDNA level [14]. Yassour et al. performed ab initio construction of the S. cerevisiae transcriptome using mRNA sequencing data [15], reporting eight genes with previously unknown splicing behavior. Of the reported cases, four splicing events modified the coding region and were capable of producing alternative protein products.

Finally, changes in the amino acid sequence by AS have been observed. Intron retention generates two different isoforms in PTC7, which determine the protein localization. The intron does not disrupt the reading frame and introduces 93 bases into the mRNA. The resulting additional 31 amino acids contain a transmembrane domain and change the localization and cellular function of the protein [16]. Usage of an alternative 5’ splice site in SRC1 introduces an earlier stop codon, shortening the protein product with functional consequences [17].

Moreover, Pelechano et al. have shown that S. cerevisiae is capable of producing a high diversity of transcript isoforms by using different transcription start and end sites. More than 26 transcript isoforms per protein-coding gene have been observed [18]. These reported cases hint at a complex transcriptional diversity in S. cerevisiae.

Intron retention, a common mechanism in fungi and plants, is often observed in S. cerevisiae splicing. Most known introns will disrupt the reading frame, leading to a premature stop codon. S. cerevisiae is able to degrade the affected transcripts using the nonsense mediated mRNA decay pathway [19], and uses this mechanism to regulate transcript levels [20]. Hossain et al. [13] demonstrate that non canonical splice signals in S. cerevisiae lead to alternative isoforms for the SUS1 gene. Furthermore, S. cerevisiae contains special factors recognizing 3’ splice sites [3] and is capable of selecting from multiple competing 3’ splice sites according to features of the pre-mRNA [21] and the presence of a uridine rich region [22]. This makes 3’ splice sites a possible target for regulated AS. In fact, the splicing factor SLU7 is known to play a role in 3’ splice site selection [23] and mutations of PRP18 have been shown to shift 3’ splice site selection in S. cerevisiae [24].

**Fig 1:**
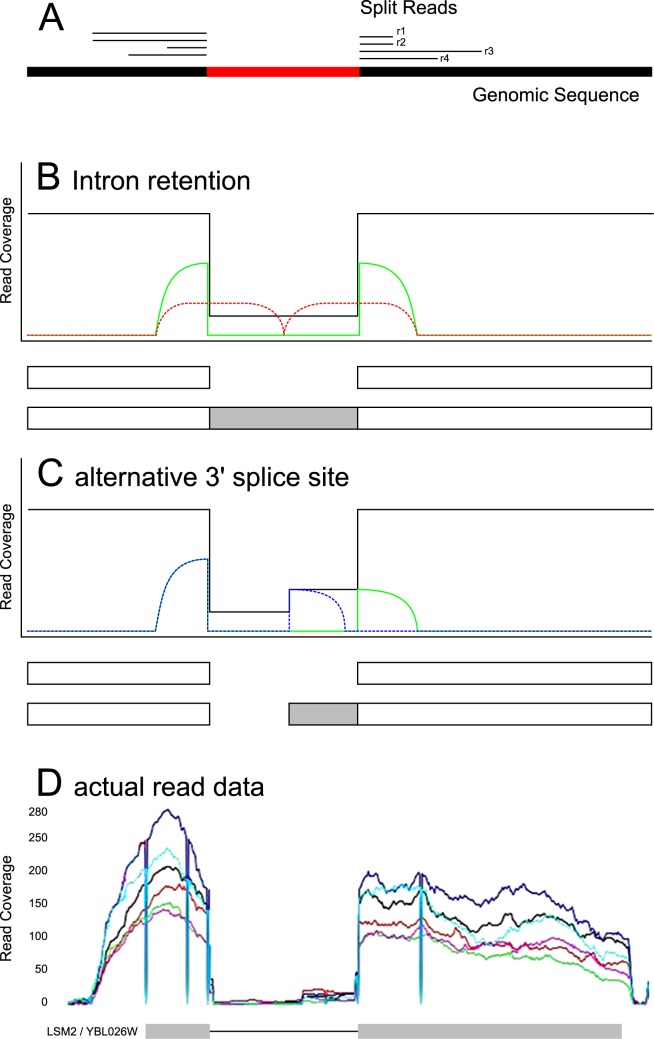
Split read mapping and AS in genes with one intron. We show the important alternative transcript models together with their idealized read coverage in next generation sequencing data. Models of all AS events described in the text are outlined in S5 Fig. The black line represents the total read coverage of all mapped reads. The green, red and blue lines represent characteristic reads for certain isoforms. **(A)** Split reads defining an intron. Reads r1 through r4 are mapped to the genome in a spliced fashion; the potential intron is supported by 4 reads. Reads r1 and r2 represent the same fragment, because they have exactly the same sequence and hence the same start and end position on the genome. As a result, the potential intron is supported by three distinct fragments. **(B)** Intron retention. The total read coverage (black) drops in the area of the intron and the intronic area is spanned by split reads (green). Ungapped (red) reads containing the 3’ or 5’ splice site are evidence of the unspliced transcript. **(C)** Alternative 3’ splice site. The total read coverage is lower in the intron, but shows an increase towards the 3’ site. There are two different types of overlapping split reads (green and blue), using the same 5’ splice site, but different 3’ splice sites. Alternative 5’ splice sites show an analogous, mirrored read coverage. Any combination of alternative 3’ and alternative 5’ splice sites is possible. **(D)** Actual read data for the gene YBL026W. The total read coverage is shown for 6 different sequencing runs (colored lines). The low read coverage within the intron and the elevated read coverage on the 3’ side of the intron are clearly visible, indicating an alternatively spliced isoform.

In this study we show that S. cerevisiae makes extensive use of its limited alternative splicing capabilities. We analyze a publicly available mRNA sequencing data set of S. cerevisiae and report novel introns, the pervasive use of alternative splice sites and evidence for unspliced transcripts that possibly occur due to intron retention. Results are validated in three different ways. Firstly by using multiple mapping programs, secondly in a complementary mRNA sequencing dataset and thirdly, via PCR, which we performed in independent S. cerevisiae samples. Advances in next generation sequencing technology provide a large number of sufficiently long reads (≥ 100 bp) that can be mapped confidently to unique positions of the genome, even if a read spans a splice junction and two ends of the read need to be mapped individually. We analyze one of the first of these available data sets by Nookaew et al. [25], observe many splice events and systematically classify them into canonical types (see Fig 1). We present the different AS event types including novel introns and discuss cases with strong evidence in the analyzed data set in detail.

## Materials and Methods

Sequencing data was obtained from Nookaew et. al. [25]. The S. cerevisiae strain CEN.PK-113-7D was used for RNA-sequencing on the Illumina platform. Total RNA was extracted from cells and purified using the RNeasy kit (Qiagen, Hilden, Germany). We followed the analysis steps from [25]. Yeast genome and annotation data was downloaded from the Saccharomyces Genome Database (SGD) [26] for reference genome R64-1-1 and from Ensembl (www.ensembl.org). Read mapping was performed using Bowtie [27] and TopHat [28] with default parameters. To ensure consistency, mapping was also performed using Star [29] and ContextMap [30]. TopHat is more conservative with respect to split read alignments (i.e. it reports less split alignments). Therefore, in the following we use the results from TopHat. The other mappings are used to cross-check the read alignments for discussed candidates. Split reads were extracted from the produced BAM-files and downstream analysis was performed with custom programs. Identified split reads are required to map unambiguously, i.e. the alignment program must not report a mapping anywhere else on the genome as continuous read.

Predicted introns were validated in another, independent next generation sequencing dataset [31]. The raw sequencing data was downloaded and mapped as described above. The resulting BAM-files were searched for split reads confirming the presence of the predicted introns under consideration. Successful validation is indicated by check marks in our tables.

Finally, several cases of novel introns and AS events are validated via PCR. Details are described in S1 Fig. In total, 3 replicates of the same PCR experiment have been performed with consistent results. Sequencing of PCR products was attempted twice, but failed in most cases due to unknown reasons. However, one example could be successfully sequenced and the predicted sequence was clearly confirmed.

### Definition of predicted introns (PIs)

For a given transcriptome sequencing data set, we define a potential intron as chromosomal subsequence which is spanned by a split read mapping, i.e. the first part of the read is mapped to the genome before the potential intron and the other part after the potential intron. Potential introns provide evidence of splicing events and, in case of overlapping potential introns, AS events. They may match or overlap known introns, according to the annotation of the Saccharomyces Genome Database, and they can also constitute novel introns.

**Fig 2:**
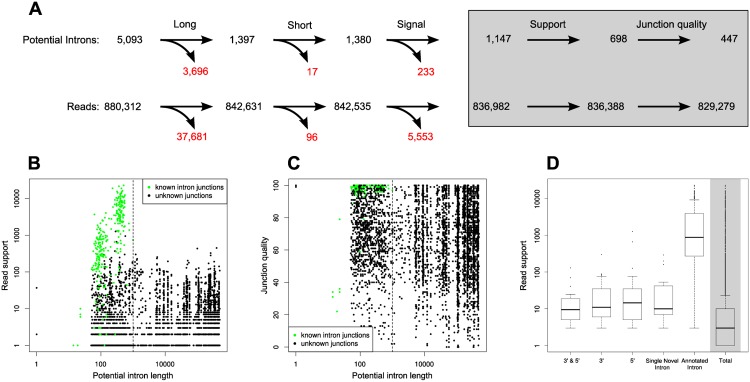
**(A)** Classification and filtering of reads and potential introns for further analysis. Long: the potential intron is longer than 1002 bases. Short: it is shorter than 40 bases. Signal: the potential intron is not flanked by a canonical splice signal. Support: the potential intron is supported by only one or two reads. After applying all filters, 94.2% of all reads (corresponding to 8.8% of all potential introns) pass this pipeline. **(B)** Read support for potential and annotated introns. The dashed line marks the longest known intron in S. cerevisiae. The high read support for annotated introns is clearly visible. There are cases of unknown potential introns with comparable read support, possibly representing novel introns. Evidence of longer introns (right of the dashed line) is not as strong, distinguishing them clearly from known introns. The two length classes of introns are visible, clustering around length 100 and 400. **(C)** Junction quality reports how well an intron junction is defined by split reads. Values close to 100 indicate long regions before and after the junction that are supported by split reads (anchor), lower values indicate shorter regions. Values also become lower if the exact split is not clearly defined, i.e. it would also map *n* bases upstream or downstream. For details refer to the methods section. **(D)** The distribution of read support for all unfiltered potential introns (total, grey background) is skewed with a median of 3 and an average of 171.1 reads. Read support for unfiltered potential introns is compared to that of filtered ones (white background): the highest read support is found among annotated introns in protein coding genes (Annotated Introns) with a median of 787 and mean of 2843.0 reads. Different classes of alternative splicing (3’, 5’, both alternative splice sites, single introns) exhibit different read support, with the lowest median (13) for combined 3’ & 5’ (both) alternative splice site usage.

We focus on potential introns that show similar characteristics to those of annotated S. cerevisiae introns in terms of length and splice signal, and which are supported by sufficient evidence. Potential introns meeting those three criteria are called predicted introns (PIs) as outlined in Fig 2a). The splice signal is defined by the two bases at the start and at the end of the potential intron. In S. cerevisiae, the majority of known introns contains the (GT—AG) splice signal. In total, we extract 6 splice signals from all annotated introns and consider those valid. The read support is the number of reads that describe the same potential intron. This number is the evidence for a predicted intron and determines the ranking we attribute to this predicted intron.

Finally, we aim to filter out the predicted introns that are not clearly defined: in the cases where the junction could also be assigned some (*n*) bases upstream or downstream due to repetitive sequence patterns at the junction boundaries, the exact position of a split is unclear. We observe that known introns are spanned by clearly defined split reads, i.e. the intron can be assigned to an exact genomic location in almost all cases. Furthermore, the supporting split reads match well before and after the junction exhibiting a long “anchor” on either side. Combining these two observations into a single score results in a measure for “junction quality”, defined as the anchor length minus *n*. Hence for each predicted intron we have two junction quality values. For PIs we require similar values as we observe for annotated introns, i.e. the maximum of both values must exceed 85 and the minimum must exceed 25 (see Fig 2c).

## Results

According to the current annotation, there are exactly 400 introns in the S. cerevisiae genome. 32 are located on the mitochondrial chromosome, 60 are annotated within transfer RNA (tRNA) and two within small nucleolar RNA. Mitochondrial introns are not considered in this study because they are spliced differently from chromosomal genes [32]; the same applies to tRNA and mitochondrial genes. Of the remaining 306 annotated introns, 282 are located within the protein coding part, and 24 are located in the 5’ UTR region. The intron length exhibits a bimodal length distribution with maxima at 100 and 400 bases [33].

With, in general, only one intron per gene, the only possible forms of AS are intron retention, alternative 3’ and alternative 5’ splice site selection. These are summarized in Fig 1. In this study we also report on “5’ UTR introns” and “novel terminal introns”. These are shown in S5 Fig. In our data, the unspliced transcript is observed for every intron containing gene, possibly due to intron retention. However, the spliced isoform always has the highest read support. Most S. cerevisiae introns will disrupt the reading frame, the resulting transcripts are thus likely to be degraded by nonsense mediated decay. In the following we focus on actual splicing events, supported by split reads.

After read mapping we obtain 880,312 mapped split reads, which define 5,093 potential introns in the genome. On average there are 173 reads per intron, but reads are distributed in such a way that a few of the potential introns are supported by a large fraction of all reads: 1,532 potential introns (30%) are supported by only one read, 2,418 by two or less reads. The median read support is 3. An overview of read support is given in Fig 2. We focus on potential introns which are likely to be a result of spliceosomal activity, so they are classified according to the properties of known S. cerevisiae introns. This way we identify “predicted introns” (PIs), which are further analyzed for their AS events. Of 5,093 potential introns, 1,147 (22.5%) remain as PIs based on splice signal and length. These are supported by 836,982 split reads (95.1%). In consequence, less than 5% of the reads support potential introns that do not show characteristics of known S. cerevisiae introns. The fraction of each classification step is shown in Fig 2a. Predicted introns with weak evidence (one or two reads) are likely to be sequencing or mapping errors and will be excluded from our further analysis. Finally, in order to ensure robustness of the results with regards to possible errors in the assignment of splice junctions during the mapping step, each predicted intron reported here must also exhibit a high junction quality. We describe this measure in detail in the methods section; roughly junction quality measures how accurately a predicted intron can be assigned to a genomic position. Filtering for low read support results in 698 predicted introns. After applying the junction quality filter to those, 447 predicted introns (8.8% of all potential introns) remain, which are supported by 829,279 reads (94.2% of all split reads).

**Fig 3:**
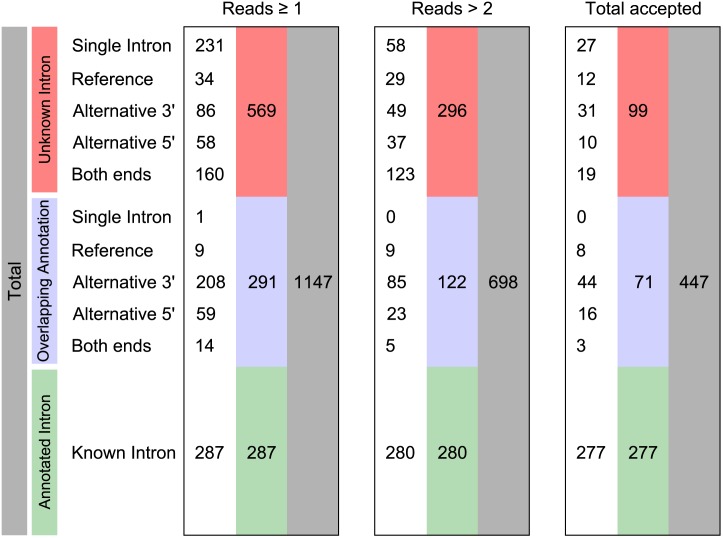
Predicted introns are assigned to different classes. “Single Introns” do not constitute evidence of AS events. “Reference” are predicted introns with further overlapping introns, i.e. AS events; the predicted intron with highest read support is selected as reference in a group of overlapping introns. The classes “alternative 3’”, “alternative 5’” and variation on “both ends” (3’ & 5’) are evidence of AS. Finally, a predicted intron may exactly overlap an annotated intron. Quantities are given for all predicted introns (reads ≥ 1, less conservative), for predicted introns with support of more than two reads, and for introns that meet all our requirements (most conservative). See also Fig 2(A).

Out of 447 predicted introns 277 match known introns. The remaining 170 predicted introns either define an alternative splice site at known introns (71 cases) or constitute novel introns (99 cases). Fig 3 shows the distribution of all predicted introns into 3’ and 5’ AS events or variation at both ends for different levels of read support. The observed AS events resulting from variation on the 3’ and 5’ splice site as well as detected novel introns are discussed below.

### Novel Introns

Out of 99 novel introns, 27 are predicted as “single intron” (see Fig 3) and 12 are predicted to have further overlapping introns, due to alternative splice site usage. Out of those, we find evidence of 8 previously unknown introns which preserve the reading frame (Table 1). In their unspliced form, the transcripts appear as annotated. In their spliced form, the resulting amino acid chains would be shortened by 6 to 23 residues. These introns are located close to the 3’ end of the gene, which is uncommon in S. cerevisiae. Only in YDR077W (SED1) is the intron located near the 5’ end of the gene. YFR031C-A (RPL2A) already contains an annotated intron, preceding the predicted intron. In each case, the annotated transcript appears to be the major isoform because the spliced isoform is supported by only a few reads.

**Table 1:** Novel introns, preserving the reading frame.

Chr.	Str.	Start	End	Length	Support	Gene
VII	-	883015	883140	126	291	YGR192C (TDH3)
VII	-	253186	253248	63	66 ✓	YGL136C (MRM2)
VI	+	107341	107442	102	50	YFL014W (HSP12)
XI	+	220778	220831	54	43 ✓	YKL117W (SBA1)
IV	+	600933	601031	99	41	YDR077W (SED1)
VIII	-	33627	33698	72	25	YHL034C (SBP1)
VII	-	437563	437628	66	23	YGL031C (RPL24A)
VI	-	220902	221108	207	13 ✓	YFR031C-A (RPL2A)

Both, the unspliced and spliced transcript isoform can yield protein products. The table shows the chromosome (Chr.), strand (Str.), Start and End of the intron, as well as intron Length, read support for the novel (i.e. spliced) isoform and the affected Gene. Check marks indicate successful validation in the independent dataset.

Four additional novel introns with sufficient read support are disruptive to the reading frame (reported in Table 2). The gene YGL030W (RPL30) contains a known intron upstream of the predicted novel intron, but there is no evidence of exon skipping. In the predicted intron we find evidence of a short variation on the 3’ splice site, shortening it by 4 bases. Genes YOR396W (YRF1-8) and YDR545W (YRF1-1) are located on different chromosomes, but share a similar sequence, therefore the novel intron appears in both genomic locations.

**Table 2:** Novel introns, disrupting the reading frame.

Chr.	Str.	Start	End	Length	Support	Gene
VII	+	439382	439479	98	56 ✓	YGL030W (RPL30)
XII	+	1073669	1073966	298	41 ✓	YLR467W (YRF1-5)
XV	+	1086634	1086931	298	36 ✓	YOR396W (YRF1-8)
IV	+	1527482	1527779	298	35 ✓	YDR545W (YRF1-1)

The unspliced transcript appears to constitute the major isoform. Splicing of the listed introns disrupts the reading frame.

Generally, for every detected intron (annotated or novel) we also find evidence of the unspliced transcript. Mostly, inclusion of annotated introns will lead to either a disrupted reading frame and/or an introduced stop codon.

### Introns in the 5’ UTR

In our data we find 56 introns located in the 5’ UTR. Among these introns, we identify 20 of 24 known introns and 36 novel introns. In Table 3 we report the novel cases with highest read support, a complete table is given as supporting information (S1 Table).

**Table 3:** Novel introns in the 5’ UTR.

Chr.	Str.	Start	End	Length	Support	Gene
XIII	-	4795	4999	205	89 ✓	YML133C
XII	+	1072192	1072397	206	76 ✓	YLR467W (YRF1-5)
XIV	-	282745	282804	60	65 ✓	YNL194C
IV	+	1526005	1526210	206	62 ✓	YDR545W (YRF1-1)
XV	+	1085157	1085362	206	62 ✓	YOR396W (YRF1-8)
II	-	5120	5335	216	50 ✓	YBL111C
II	+	691967	692133	167	43 ✓	YBR237W (PRP5)
XIII	+	610808	611036	229	41 ✓	YMR175W (SIP18)

### 3’ and 5’ splice site variation

Under the most conservative assumptions we identify a total of 63 annotated introns with variable splice sites. These divide into 44 cases of an alternative 3’ splice site, 16 cases of an alternative 5’ splice site and three cases with combined alternative 3’ & 5’ splice sites. There are 22 (3’) and 8 (5’) cases with 10 or more supporting split reads. Compared to 3’ and 5’ alternative splice sites, combined alternative splice sites exhibit the lowest read support (see Fig 2d).

Among the alternative 3’ splice sites the median distance to the canonical splice site is 28 bases. There are 11 cases that do not disrupt the reading frame and are able to encode an altered protein product. The AS events are able to remove or introduce between 1 and 53 amino acids compared to the reference sequence. The remaining 33 cases will disrupt the reading frame, if the intron is located in the protein coding region. Table 4 lists the highly supported cases that preserve the reading frame and Table 5 lists the highly supported examples of introns that are disruptive to the reading frame. A complete list is given in S1 Table.

**Table 4:** Alternative 3’ splice sites preserving the reading frame.

Chr.	Str.	Start	End	Distance	Support	Gene
XVI	-	5778	5988	+63	203 ✓	YPL283C (YRF1-7)
XI	+	155272	155636	-18	86 ✓	YKL157W (APE2)
IV	+	652781	653526	+3	81 ✓	YDR099W (BMH2)
XIII	+	732466	733034	+159	39 ✓	YMR230W (RPS10B)
VIII	-	498708	498786	+12	35 ✓	YHR199C-A (NBL1)
II	+	393181	393507	-3	28 ✓	YBR078W (ECM33)
XI	-	93303	93465	+9	24 ✓	YKL186C (MTR2)
XII	+	1067085	1067303	-60	22 ✓	YLR464W
II	+	653369	653524	+72	16 ✓	YBR215W (HPC2)

The table shows the chromosome (Chr.), strand (Str.), Start and End of the alternative intron, the Distance between the alternative splice site and the canonical splice site, the read Support of the isoform and the affected Gene. Check marks indicate successful validation in the independent dataset.

**Table 5:** Alternative 3’ splice sites with potential to disrupt the reading frame.

Chr.	Str.	Start	End	Distance	Support	Gene
Introns in the protein coding region:						
II	+	170677	170757	47	81 ✓	YBL026W (LSM2)
XI	-	93367	93465	55	55 ✓	YKL186C (MTR2)
IV	-	254956	255044	19	40 ✓	YDL115C (IWR1)
V	-	269784	270148	32	25 ✓	YER056C-A (RPL34A)
Introns in the 5’ UTR (reading frame irrelevant):						
XII	+	855878	856427	7	300 ✓	YLR367W (RPS22B)
X	-	172413	172752	20	72 ✓	YJL130C (URA2)
XVI	+	115219	115298	5	42 ✓	YPL230W (USV1)

Examining some of those cases in more detail, we find examples in which the AS event may trigger well known biological functions: Alternative splicing in YDR099W (BMH2) and YBR078W (ECM33) introduces a single additional codon, the splice site is of the NAGNAG form, providing two adjacent acceptor sites. The additional codon introduces a new amino acid into the resulting protein sequence, which has been reported as a mechanism to control protein localization [34] and as a conserved form of regulated AS in higher eukaryotes [35]. Genes YPL283C (YRF1-7), YPR202W, YNL339C (YRF1-6) and YGR296W (YRF1-3) share almost identical sequences, so the splicing pattern is predicted to be the same for those genes. In the gene YKL157W (APE2) the annotated intron is shortened by 18 bases and the putative alternative splice site is located closer to the present TACTAAC branch point sequence.

The remaining alternative 3’ splice sites are located downstream of the canonical splice site, extending the intron and removing amino acids from the protein product. Intron extension may be explained by a variation in the linear scanning mechanism proposed for detection of the splice acceptor site starting from the branch point [22].

### Novel terminal introns

We find evidence of three cases of novel introns overlapping the genes 3’ end (Table 6). Splicing of the exon leads to removal of the stop codon, extending the transcript to an alternative downstream stop codon. This results in alternative C-terminal amino acid sequences of the translated protein or, in one observed case, to the fusion of the transcript with the next downstream transcript on the genome.

**Table 6:** Novel introns containing the annotated stop codon.

Chr.	Str.	Start	End	Length	Support	Gene
II	-	443706	443833	128	200 ✓	YBR101C (FES1)
XIII	+	559783	560157	375	162 ✓	YMR147W
VII	+	436313	436374	62	53 ✓	YGL033W (HOP2)

The table shows the chromosome (Chr.), strand (Str.), Start and End position of the novel intron, as well as the intron Length, the read Support of the spliced isoform and the affected Gene. Check marks indicate successful validation in the independent dataset.

In the spliced isoform of YBR101C (FES1) the terminal YVL* is substituted with TFCKMEFIKKVRRGK*. In case the terminal intron in YMR147W is spliced, the alternative protein terminus extends into the reading frame of YMR148W (OSW5), leading to a chimeric protein fusion. This confirms a previously described observation [14]. The intron spanning the stop codon in YGL033W (HOP2) is also responsible for two different C-terminal peptide sequences: the unspliced, annotated stop codon results in the amino acid sequence VCILNIFRDLFF*, while the skipped stop codon results in the shorter EEIGFEDI*.

### Validation of alternative transcripts

The alternative splicing events reported in this paper were validated in three different ways: (A) by using different mapping software, (B) in an independent sequencing dataset, and (C) via PCR for selected splicing events.

(A) The first validation was performed using two other mapping programs (Star [29] and ContextMap [30]) on the original data. All split read mappings were also reported by at least one other program, with the exception of the novel intron predicted in YDR077W (SED1) (See Table 1). The alternative splicing events reported in S1 Table were also checked and more than 95% were consistently mapped by at least one other program.

(B) Predicted introns were cross checked against read data from another next generation sequencing dataset [31]. We consider a predicted intron independently validated, if we find its respective split reads in the second dataset as well. Successful validation of predicted introns is indicated by check marks in Tables 1 through 6. Out of 39 splicing events, comprising novel introns and AS events, 34 can be validated in the second dataset.

(C) We used S. cerevisiae samples to validate our identified splicing and alternative splicing events by PCR. Primers could be designed if the following conditions were met: First, the length difference of the expected PCR products had to be large enough, such that different products would become visible on the gel. Second, the gene and isoform under consideration had to be expressed in our available S. cerevisiae samples. Primers must be unique and close enough to the alternative event and we require them to lie within genomic regions that are transcribed in the used samples. We also designed primers for three additional cases in which we found striking evidence of alternative splicing or splicing in our own S. cerevisiae samples. These cases are also found in our original dataset [25], but with weak evidence (S1 Table).

Under the abovementioned constraints, we designed primer pairs for 10 predicted novel introns: Five pairs were designed for novel introns in the 5’ UTR, three pairs for novel introns in protein coding regions and two pairs for alternative 3’ splice sites at two known introns. Out of those 10, PCR confirmed alternative splicing in 7 cases: splicing was confirmed for the 5’ UTR introns at genes YLR467W (YRF1-5), YBL111C and YGL063W (PUS2) (in the remaining two, YFL064C and YDR545W, we could not confirm splicing). Furthermore, AS could be confirmed for the novel introns in YBR101C (FES1), and in YMR147W. In both cases the annotated stop codon is observed as both, included and spliced out in the isoform. The third novel intron in the protein coding region of YGL136C (MRM2) is alternatively spliced according to the sequencing data, and PCR confirms the presence of an intron. Finally, PCR confirmed the alternative 3’ splice site in YBL026W (LSM2), but not in YOR293W (RPS10A). Pictures of the gels and sequences of all primers are listed in the supporting information (S1 Fig and S2 Table).

## Discussion

We analyzed alternative splicing (AS) in the yeast S. cerevisiae. High throughput mRNA sequencing data with sufficient read length enable us to confidently map split reads to unique positions on the genome. As a result we can identify an unprecedented amount of previously unknown introns and observe several cases of alternative intron usage in S. cerevisiae. Some of these AS events have been described earlier and are referenced in the introduction; in most of these cases the alternative isoform shows high read support in our analyzed data and may be detected more easily in general. In addition to the previously described AS events we find novel cases, indicating a more widespread use of AS in S. cerevisiae than currently known. Strikingly, we validate most of the reported alternative events in a different, independent NGS dataset. We then validate reproducible splicing events via PCR in 7 out of 10 tested cases in a dedicated experiment.

In about 20% of annotated introns we find evidence of alternative 3’ or 5’ splice sites, with a strong bias toward 3’ variation. Compared to 5’ splice sites, S. cerevisiae exhibits weaker splice signals at 3’ splice sites, which facilitates alternative splicing [2]. The slackness of 3’ splice site selection may account for the remarkably higher variation at this end. This mechanism appears to be widely used by S. cerevisiae and can beneficially introduce or excise small peptide sequences from the resulting proteins. Another mechanism to alter the protein sequence, specifically on the C-terminal end, are introns removing the stop codon. This leads to an alternative stop codon or, in one observed case, to the fusion of two coding regions into one reading frame.

Reads supporting intron retention can be found in every intron containing gene, albeit with low read support in general. The mechanism is common in fungi and plants and serves regulatory purposes, but the retained introns could also be an artifact of the stochastic nature of the splicing process. As such they might be yet unspliced transcripts or defective splicing, which will be targeted by NMD. However, we find evidence of eight novel introns that are not disruptive to the reading frame. The relatively weak evidence for the spliced, novel isoforms could explain why these introns have not been identified earlier. Intron retention has been shown to cause alternative proteins in at least one S. cerevisiae gene (PTC7) [16]), where the annotated, spliced transcript retains its intron and constitutes a novel protein product. Here we observe the complementary case: the annotated, continuous transcript is spliced and some sequence removed. The newly detected introns might appear due to very weak splicing signals, but the predicted novel isoforms could be translated and functional. Only targeted experiments can resolve whether this is indeed the case.

There are 10 annotated cases of multi intron genes, and we predict a second, novel intron in the gene YFR031C-A (RPL2A). The respective genes contain two introns each and allow the possibility of AS induced exon skipping or cassette exons. Exon skipping has been reported for the SUS1 gene [13], but in our data we observed no evidence thereof, neither in SUS1 nor any other multi intron gene.

We conservatively analyzed a single dataset with the hypothesis of limited AS in S. cerevisiae. We used a small dataset that comprises two conditions and systematically screened for variation among annotated introns. Nevertheless, we detect novel alternative splicing events that are subsequently validated using independent data. In general the predicted novel introns are supported by less reads than annotated introns. We conclude that the respective transcripts are minor isoforms, possibly triggered by specific conditions. They may coexist with the major isoform and are therefore rather hard to detect. PCR of alternative events resulted in the successful validation of most of the tested isoforms. While isoforms with weak evidence should be considered with care or examined under different experimental conditions, we suggest that highly supported alternative isoforms are more likely functional. This observation is supported by recent work of Kawashima et al. [4], in particular as the NMD pathway has been demonstrated to effectively remove aberrant AS products. Comparing the support of isoforms with high read support in wild type S. cerevisiae to NMD defective strains shows that there are novel AS isoforms which are not targeted by NMD (see S3 Fig). The role of other nuclear RNA decay pathways [36] on alternative transcripts in S. cerevisiae would be an interesting addition to that work.

Kawashima et al. also publish a list of 728 splicing events, of which 522 suggest alternative splicing. However, the authors do not discuss them in detail. The overlap between our study and Kawashima et al. is not expected to be high, since our analysis pipeline is different (e.g. Kawashima et al. also report alternative splice sites without canonical splice signals) and AS is likely to be context specific. Consequently, differences in yeast strain and growth conditions result in different AS. Furthermore, technical factors like NGS library preparation method and sequencing depth and length will influence the detection of isoforms in each study. The overlap between 170 potential introns in our study and the 522 AS events is 13. Refer to S2 Fig for a more detailed view. Another study by Volanakis et al. [5] reports 78 splicing events, but they do not overlap with our AS events.

In conclusion, AS in S. cerevisiae is possible and observed on the transcript level. Using split reads in mRNA-sequencing data we identify several cases of alternative transcripts, which can be explained by an alternative splicing mechanism in S. cerevisiae. Systematic analysis of AS events will help to investigate the impact on the proteome and to identify the regulatory mechanisms leading to isoform production. AS in S. cerevisiae is triggered by conditions like elevated heat [16] or restrictive growth conditions. Since we analyzed high throughput data for a small subset of all conceivable conditions, we expect to observe only a tiny fraction of the AS capabilities of S. cerevisiae in this study. Integrating more extensive forthcoming data on more conditions using our approach (e.g. [31]), will enhance our understanding of AS in S. cerevisiae.

## Supporting Information

S1 Table Predicted Introns. List of all predicted novel introns, sorted by evidence (read support). For completeness we also report potential introns with a read support lower than three. Columns include Chromosome, Strand, Start, End, Length specifying the position of the novel predicted intron. Additional columns are
Read Support: evidence for the predicted intron.
Anchor—Slack: junction quality scores.
Gene: affected gene.
PCR: if PCR has been performed, capital letters identify the lane in S1 Fig.
(PDF)

S1 Fig PCR gels of tested predicted introns. PCR gels of 10 tested novel introns and alternative splicing events. The expected position of PCR products confirming novel events are marked with arrows. Numbers describe the adjacent PCR product size (in bases) of the two marker bands. Chromosomal locations of the events are listed below.
The S. cerevisiae strain BY4741 (MATa; his3 Δ1; leu2 Δ0; met15Δ0; ura3Δ0) (Euroscarf, Frankfurt, Germany) was cultivated in YPD at 30°C. Exponentially growing S. cerevisiae cells at an OD595 of 0.8 were collected and used for preparation of RNA applying the SV Total RNA Isolation System (Promega, Madison, USA). For first strand cDNA preparation 1 *μ*g of total RNA was transcribed in a 25 *μ*l reaction using the M-MLV reverse transcriptase (Promega, Madison, USA) according to manufacturer instructions. 1 *μ*l of the resulting first strand cDNA was used as template in 50 *μ*l PCR reactions (0.2 mM dNTPs; 1 *μ*M primer; 1.5 mM MgCl2; 35 cycles using an annealing temperature of 55°C and 1min synthesis time at 72°C) applying splice variant specific primer pairs (S2 Table) and GoTaq G2 DNA Polymerase (Promega, Madison, USA). 10 *μ*l of the PCR reactions were analyzed on 2% agarose gels stained with DNA Stain Clear G (Serva, Heidelberg, Germany).
The above experiment was repeated three times. The first time without any sequencing attempt. The second time sequencing failed. The third time sub-clonig and subsequent sequencing was done:
For sub-cloning, 50 µl PCR reactions were completely separated on 2% agarose gels and the bands corresponding to the splice product were purified using the MiniElute PCR Purification Kit (Qiagen, Hilden, Germany). The purified splice product was sub-cloned using the TOPO TA Cloning Kit for Sequencing (Life Technologies, Carlsbad, USA) according to the manufacturer’s protocol. Positive clones were selected after DNA-preparation by control PCRs with the respective primer set and further analyzed by sequencing (MWG Eurofins, Ebersberg, Germany).
In Experiment J the “expected novel fragment length” could be sequenced and is matching the expected sequence. This coincides with the best visible PCR band out of any of the “expected novel fragment length” bands.
(PDF)

S2 Table Primer pairs. Location and sequences of the PCR primers used.
(PDF)

S2 Fig Overlap with published data. We compare our results with the recently published results of Kawashima et al. Venn diagrams compare potential intron assignments at two different levels of confidence in our analysis to introns reported by Kawashima et al. In each plot the set “All” contains all introns fulfilling our length requirements and presenting a valid splice signal. The set “Only accepted” only contains introns that additionally fulfill our support and junction quality criteria.
A) Overlap limited to annotated introns. We observe a large overlap of about 90% introns reported in Kawashima et al. [4] and about 2/3 of our introns.
B) Overlap without annotated introns. In these diagrams we compare true alternative splicing events reported here and by Kawashima et al. The overlap among proposed isoforms is not expected to be high, since our analysis pipeline is different (e.g. Kawashima et al. also report alternative splice sites without canonical splice signals) and AS is likely to be context specific. Some isoforms are exactly found in both studies, so those results are reproducible under different experimental and technical conditions.
c) Out of the highly confident AS events reported in the tables in the main text, five are also reported by Kawashima et al. Strikingly, all of them are at the 3’ splice site, and four preserve the reading frame. The set “Reported” only contains the predicted introns that are listed in the tables in this manuscript excluding any novel introns (n = 20), because those are not considered by Kawashima et al.
The potential introns in the sets “All” and “Only accepted” are derived following our workflow, depicted in Fig 1 in the manuscript. Summing up “All” (107 + 180 + 804 + 56) results in 1,147 potential introns and summing up “Only accepted” (103 + 174 + 157 + 13) results in 447 predicted introns. (These numbers also appear in Figs 2(A) and 3)
(PDF)

S3 Fig Nonsense mediated decay (NMD) of highly supported isoforms. The data published by Kawashima et al. [4] enables us to compare the support of isoforms with high read support in wild type (WT) S. cerevisiae to NMD defective (knockout) strains. To this end, we mapped the data by ourselves and analyzed the results. In the boxplots outliers are removed, and mean values are given as additional information (red numbers).
In summary, we confirm the findings of Kawashima et al. Additionally, we detect some unknown AS events that seem to be unaffected by NMD:
Panel A shows how the split read support for annotated introns changes between WT and the three NMD defective strains (d1, d2, d3). Note that annotated introns have a higher median and mean read support in WT, than in any knockout strain. This changes among the unannotated introns in Panel B.
Panel B shows the read support for predicted, unannotated introns. By requiring read support in at least two knockout strains, we gain confidence for each predicted intron. The negative fold change from knockout to WT is a result of efficient NMD in WT. This confirms the findings of Kawashima et al.
In panel C we show all the predicted introns that are supported by at least two reads in WT, and by at least one read in one of the knockout strains. As a result, we are confident about the predicted introns in WT and able to compare them to the knockout strains. The boxplot clearly shows that the lack of NMD (in the knockout strains) has little or no effect on isoforms that are confidently expressed in WT. Consequently, such isoforms are unlikely targets of NMD in WT and, therefore, more likely functional. Also, there are other nuclear RNA decay pathways than NMD that might target those transcripts.
Scatter plot D shows the same data as box plot B (including outliers): for each predicted intron, the WT read support is plotted against the mean read support of the knockout strains. In particular, the predicted introns with high support in WT do not show a significant fold change, so those transcript isoforms are not depleted in wildtype S. cerevisiae and, therefore, unlikely NMD targets.
Unfortunately, a large scale comparison of the predicted isoforms based on our original dataset is not feasible, because the overlap of isoforms between the different datasets is not sufficiently large (see S2 Fig).
(PDF)

S4 Fig Rate of validation in an independent dataset. Some additional evidence for a predicted intron would be its validation in a different, independent dataset. We successfully validated the most confident intron predictions by detecting their split reads in data from Waern and Snyder [31]. In this figure we quantitatively compare the validation of potential introns by their read support in our original dataset (Nookaew et al. [25]) and the independent dataset (Waern and Snyder). Reads are pooled across experimental conditions and replicates.
Figure on top: Evidently, annotated introns show high read support in either dataset and their read support seems to be correlated among datasets (Pearson’s *ρ* = 0.71). Among the unknown splice junctions (red circle), the correlation drops (*ρ* = 0.43); finally, those with a non-canonical splice signal (blue x) tend toward lower read support in general and the read support appears not to be correlated (*ρ* = −0.01).
In the figure at the bottom we additionally show potential introns that do not pass our length filter (green x). The majority of those long introns shows a low read support and no correlation among datasets (*ρ* = 0.16), qualifying them as possible read or mapping errors. However, there are some potential introns covering long stretches of the S. cerevisiae genome. Some of these can also be validated in the independent dataset (green crosses around the center of the plot). These read mappings can either be explained by genomic rearrangements in the used strains or point toward post-transcriptional mechanisms in S. cerevisiae. Since there is no known mechanism explaining such transgenic splicings, the presence of these transcriptional products needs to be validated by other means.
(PDF)

S1 File Hypothetical protein sequences of genes with predicted introns. Some predicted isoforms might be translated into functional protein isoforms. In this file we list an in silico translation of all S. cerevisiae genes for which we predict alternative isoforms. The sequences might aid further research on the respective genes. In each record, the first line reports the gene along with the genomic location. The next line reports the predicted intron, followed by all annotated introns of the gene. Next, the length difference of the annotated and the predicted alternative transcript is given in bases: negative values indicate deletion and positive values indicate insertion events. Finally, the transcript and translated protein sequence are reported for the gene as annotated (ORI) and for the predicted isoform (VAR).
(TXT)

S5 Fig Diagrams for each class of novel introns and alternative splicing described in the text. Alternative transcript models together with their idealized read coverage in next generation sequencing data. The black line represents the total read coverage of all mapped reads. The green, red and blue lines represent characteristic reads for certain isoforms. **(A)** Split reads defining an intron. Reads r1 through r4 are mapped to the genome in a spliced form; the potential intron is supported by 4 reads. Reads r1 and r2 represent the same fragment, because they have exactly the same sequence and hence the same start and end position on the genome. As a result, the potential intron is supported by three distinct fragments. **(B)** Intron retention. The total read coverage (black) drops in the area of the intron and the intronic area is spanned by split reads (green). Ungapped (red) reads containing the 3’ or 5’ splice site are evidence of intron retention. **(C)** Alternative 3’ splice site. The total read coverage is lower in the intron, but shows an increase towards the 3’ site. There are two different types of overlapping split reads (green and blue), using the same 5’ splice site, but different 3’ splice sites. **(D)** Alternative 5’ splice site. Similar to alternative 3’ splice sites, but mirrored. **(E)** 5’ UTR intron. The intron is located before the start codon and the coding sequence (yellow) starts downstream. **(F)** Novel terminal intron. The intron overlaps the annotated stop codon and splices it out, extending the coding sequence to the next downstream stop codon.
(PDF)
