# Supplementary material for: Alternative Splicing in Next Generation Sequencing Data of Saccharomyces cerevisiae
Source: PLoS One. 2015 Oct 15;10(10):e0140487. doi: 10.1371/journal.pone.0140487 (PMC4607428; doi:10.1371/journal.pone.0140487)
Supplement: S1 Table — List of all predicted novel introns, sorted by evidence (read support). For completeness we also report potential introns with a read support lower than three. Columns include Chromosome, Strand, Start, End, Length specifying the position of the novel predicted intron. Additional columns are Read Support: evidence for the predicted intron. Anchor—Slack: junction quality scores. Gene: affected gene. PCR: if PCR has been performed, capital letters identify the lane in S1 Fig. (PDF) [file pone.0140487.s001.pdf]

Novel Introns in the 5' UTR

Chromosome	Strand	Start	End	Length	Read support	Anchor – Slack 1	Anchor – Slack 2	Gene	PCR
XII	-	464709	465069	361	96	98	97	YLR154C-G	
XIII	-	4795	4999	205	89	94	99	YML133C	
XII	+	1072192	1072397	206	76	90	96	YLR467W	D
IX	+	246216	246553	338	74	97	91	YIL060W	
XIV	-	282745	282804	60	65	91	52	YNL194C	
IV	+	1526005	1526210	206	62	90	96	YDR545W	C
XV	+	1085157	1085362	206	62	90	96	YOR396W	
II	-	5120	5335	216	50	94	91	YBL111C	E
II	+	691967	692133	167	43	43	92	YBR237W	
XIII	+	610808	611036	229	41	66	98	YMR175W	
VIII	+	557497	557712	216	37	90	90	YHR218W	
XII	-	4919	5123	205	35	92	99	YLL067C	
IX	-	6258	6468	211	30	92	99	YIL177C	
XII	-	10454	10658	205	29	92	99	YLL066C	
V	-	5824	6010	187	28	92	99	YEL075C	
VIII	-	4652	4857	206	27	96	34	YHL049C	
XII	+	64132	64359	228	25	58	93	YLL037W	
VII	+	383486	383565	80	22	70	96	YGL063W	G
VI	-	3957	4167	211	20	95	64	YFL064C	B
X	-	6241	6451	211	20	92	76	YJL225C	
XII	-	464319	464550	232	17	100	100	YLR154C-G	
II	+	407024	407118	95	17	24	99	YBR083W	
XII	-	4939	5123	185	12	83	67	YLL067C	
VIII	+	548712	548825	114	12	92	-45	YHR215W	
XII	-	465652	465715	64	8	100	86	YLR154C-G	
IX	-	6278	6468	191	8	85	99	YIL177C	
V	-	5844	6010	167	8	85	99	YEL075C	
XIII	-	4815	4999	185	8	83	99	YML133C	
II	+	691967	692125	159	8	24	91	YBR237W	
VIII	-	103618	103856	239	6	93	68	YHL002C-A	
XII	+	1065659	1065845	187	5	90	14	YLR462W	
IV	+	1063188	1063340	153	5	64	92	YDR301W	
VII	+	383486	383580	95	4	71	96	YGL063W	
VII	+	437938	438393	456	3	90	11	YGL030W	
II	+	342699	342806	108	2	35	100	YBR054W	
XV	-	18291	18826	536	2	69	95	YOL159C	

3' alternative splice site, non-disruptive to reading frame

Chromosome	Strand	Start	End	Length	length difference t	Read support	Anchor – Slack 1	Anchor – Slack 2	Gene	
XI	+	155272	155636	365	18	86	97	87	YKL157W	
IV	+	652781	653526	746	3	81	87	94	YDR099W	
XIII	+	732466	733034	569	159	39	92	96	YMR230W	
VIII	-	498708	498786	79	12	35	88	93	YHR199C-A	
II	+	393181	393507	327	3	28	88	96	YBR078W	
II	+	653369	653524	156	72	16	87	86	YBR215W	
II	-	186266	186427	162	87	14	70	79	YBL018C	
VIII	+	126552	127343	792	231	9	53	94	YHR010W	
XVI	+	173666	174081	416	9	9	78	95	YPL198W	
IV	+	122078	122194	117	18	6	71	83	YDL189W	
XV	+	867150	867589	440	3	6	65	90	YOR293W	
VII	-	787784	788178	395	3	5	91	31	YGR148C	
XII	+	744154	744239	86	48	5	79	66	YLR306W	
VIII	+	251156	251458	303	210	5	79	69	YHR076W	
XV	+	867150	867745	596	159	5	67	96	YOR293W	A
VII	+	62132	62183	52	6	4	48	49	YGL232W	
XII	+	987142	987221	80	9	3	65	73	YLR426W	
IV	+	652781	653562	782	39	3	73	70	YDR099W	
IV	+	652781	653490	710	33	3	58	57	YDR099W	
XIII	+	732466	732911	446	36	3	55	45	YMR230W	
II	-	679928	680039	112	15	3	93	23	YBR230C	
II	+	604515	604930	416	3	3	37	75	YBR189W	
XV	+	867150	867622	473	36	3	77	36	YOR293W	
VI	-	64588	64920	333	12	3	52	34	YFL034C-A	
VI	-	64354	64920	567	246	3	82	27	YFL034C-A	
XII	+	987142	987233	92	21	2	43	59	YLR426W	
IX	-	4607	4986	380	81	2	93	62	YIL177C	
IV	-	1319748	1319816	69	27	2	52	36	YDR424C	
IV	+	1103810	1104732	923	840	2	45	53	YDR318W	
IV	+	399362	400141	780	657	2	28	28	YDL029W	
IV	+	122078	122167	90	9	2	33	54	YDL189W	
V	+	131777	131902	126	3	2	44	63	YEL012W	
XIII	-	140079	140183	105	12	2	73	28	YML067C	
III	-	101643	101700	58	9	2	67	95	YCL012C	
II	+	462430	462559	130	60	2	49	61	YBR111W-A	
VI	-	64357	64920	564	243	2	38	40	YFL034C-A	
VII	-	497893	497999	107	45	1	67	31	YGR001C	
VII	-	497339	497458	120	27	1	66	35	YGR001C	
VII	-	72968	73137	170	21	1	30	68	YGL226C-A	
IX	-	99069	99385	317	27	1	44	22	YIL133C	
XI	+	551681	552053	373	51	1	29	71	YKR057W	
XII	+	786616	786701	86	18	1	50	42	YLR329W	
XI	+	166400	166535	136	48	1	24	63	YKL150W	
IV	-	1319609	1319697	89	9	1	65	11	YDR424C	
IV	+	1103810	1103907	98	15	1	47	43	YDR318W	
IV	+	652781	653640	860	117	1	43	42	YDR099W	
IV	+	629906	630056	151	117	1	42	55	YDR092W	
IV	+	629906	630041	136	132	1	37	60	YDR092W	
IV	+	337525	337880	356	246	1	66	31	YDL064W	
IV	+	322283	322724	442	21	1	50	51	YDL075W	
IV	+	122078	122206	129	30	1	50	43	YDL189W	
XII	+	242322	242992	671	312	1	24	72	YLR048W	
V	-	269788	270148	361	36	1	58	42	YER056C-A	
V	+	548553	548632	80	12	1	44	51	YER179W	

X	-	396467	396570	104	27	1	48	46	YJL024C
X	-	50344	50411	68	75	1	74	26	YJL205C
X	+	435228	435763	536	420	1	22	53	YJL001W
X	+	435228	435316	89	27	1	71	22	YJL001W
X	+	91095	91417	323	6	1	22	76	YJL177W
XIII	-	721115	721345	231	84	1	53	44	YMR225C
XIII	-	123701	124157	457	42	1	67	29	YML073C
VIII	-	315733	315858	126	39	1	34	65	YHR101C
II	-	110220	110505	286	201	1	51	35	YBL059C-A
II	-	60209	60697	489	15	1	65	36	YBL087C
II	+	653369	653539	171	87	1	33	60	YBR215W
II	+	393181	393669	489	159	1	49	47	YBR078W
II	+	125155	125282	128	12	1	36	64	YBL050W
XVI	+	406647	407019	373	48	1	29	36	YPL079W

3' alternative splice site, disruptive to reading frame

Chromosome	Strand	Start	End	Length	length difference t	Read support	Anchor – Slack 1	Anchor – Slack 2	Gene	
XII	+	855878	856427	550	7	300	63	98	YLR367W	
II	+	170677	170757	81	47	81	96	92	YBL026W	F
X	-	172413	172752	340	20	72	74	98	YJL130C	
XVI	+	115219	115298	80	5	42	56	87	YPL230W	
IV	-	254956	255044	89	19	40	94	95	YDL115C	
V	-	269784	270148	365	32	25	94	45	YER056C-A	
XI	-	93303	93465	163	64	24	82	99	YKL186C	
XIV	+	350958	351060	103	7	23	74	76	YNL147W	
III	-	111580	111633	54	22	20	95	68	YCL002C	
VII	+	1084883	1085073	191	20	18	98	96	YGR296W	
XIV	-	5889	6079	191	20	18	95	99	YNL339C	
XVI	-	5798	5988	191	20	18	95	99	YPL283C	
IV	+	1359970	1360373	404	31	17	51	67	YDR450W	
V	-	4383	4601	219	173	17	64	98	YEL076C-A	
VI	-	64592	64920	329	8	17	92	73	YFL034C-A	
I	-	150990	151096	107	17	16	78	75	YAL001C	
III	-	101606	101700	95	28	14	98	85	YCL012C	
VII	+	62132	62196	65	7	12	64	79	YGL232W	
XII	-	931336	931698	363	14	12	95	86	YLR406C	
XII	-	327255	327399	145	4	10	63	77	YLR093C	
IV	-	733686	733775	90	17	10	83	97	YDR139C	
VIII	+	251156	251258	103	10	10	73	69	YHR076W	
VII	+	920665	921129	465	10	8	73	98	YGR214W	
IV	+	122078	122186	109	10	8	82	92	YDL189W	
XIII	+	424998	425124	127	29	8	39	81	YMR079W	
VII	+	727039	727385	347	28	7	67	57	YGR118W	
IX	-	99131	99385	255	35	7	90	54	YIL133C	
IV	-	254943	255044	102	32	6	65	97	YDL115C	
II	-	142754	142846	93	4	6	89	71	YBL040C	
VII	-	72985	73137	153	4	5	70	40	YGL226C-A	
IX	+	47699	47764	66	4	5	50	97	YIL156W-B	
XI	+	551681	552043	363	41	5	29	97	YKR057W	
XI	+	155272	155629	358	25	5	81	73	YKL157W	
XII	+	242322	242690	369	10	5	79	81	YLR048W	
XVI	+	943051	943241	191	20	5	71	89	YPR202W	
XI	+	166400	166516	117	29	4	73	71	YKL150W	
IV	+	1359970	1360438	469	34	4	51	98	YDR450W	
IV	+	579479	580024	546	7	4	31	83	YDR064W	
XIII	-	666923	667017	95	11	4	59	69	YMR201C	
XIII	-	223238	223781	544	143	4	96	61	YML026C	
VIII	-	138237	138408	172	4	4	61	84	YHR016C	
II	+	462210	462269	60	20	4	62	66	YBR111W-A	
XII	+	987142	987349	208	137	3	42	56	YLR426W	
XI	+	551681	552022	342	20	3	40	98	YKR057W	
XI	+	109578	109966	389	83	3	98	44	YKL180W	
XI	+	82999	83081	83	7	3	38	84	YKL190W	
IV	+	1401801	1402404	604	220	3	20	98	YDR471W	
IV	+	1236843	1237630	788	22	3	39	76	YDR381W	
IV	+	652781	653555	775	32	3	53	75	YDR099W	
XII	+	242322	242826	505	146	3	44	85	YLR048W	
XIV	+	557611	557698	88	14	3	76	63	YNL038W	
XIV	+	185492	185578	87	8	3	30	89	YNL246W	
V	+	131777	131880	104	19	3	72	37	YEL012W	
X	-	156770	157249	480	20	3	84	36	YJL136C	
XIII	-	223235	223781	547	146	3	94	41	YML026C	
III	-	101585	101700	116	49	3	55	69	YCL012C	
III	+	293940	294007	68	14	3	17	85	YCR097W	
VIII	-	505244	505516	273	4	3	96	30	YHR203C	
VIII	-	298381	298484	104	20	3	90	52	YHR097C	
XVI	-	729354	729481	128	32	3	74	56	YPR098C	
VI	-	54376	54686	311	2	2	60	75	YFL039C	
VII	-	497349	497458	110	17	2	40	95	YGR001C	
VII	-	157232	157282	51	32	2	58	37	YGL183C	
VII	+	1084883	1085053	171	40	2	40	63	YGR296W	
VII	+	727039	727596	558	239	2	72	38	YGR118W	
XI	+	625902	625986	85	10	2	61	54	YKR095W-A	
IV	+	1103810	1103881	72	11	2	54	86	YDR318W	
IV	+	217603	218117	515	110	2	26	72	YDL136W	
XIV	-	5909	6079	171	40	2	59	43	YNL339C	
X	-	50322	50411	90	53	2	83	26	YJL205C	
XIII	-	666786	667017	232	148	2	68	36	YMR201C	
XIII	-	140074	140183	110	17	2	82	28	YML067C	
XIII	+	732466	732844	379	31	2	71	42	YMR230W	
II	-	726826	727011	186	92	2	56	51	YBR255C-A	
II	-	186336	186427	92	17	2	42	57	YBL018C	
II	-	142728	142846	119	22	2	51	48	YBL040C	
II	+	653369	653490	122	38	2	27	74	YBR215W	
II	+	170677	170844	168	40	2	73	32	YBL026W	

II	+	125155	125317	163	47	2	97	25	YBL050W	
XV	+	505938	506030	93	308	2	22	87	YOR096W	
XVI	-	492948	493020	73	29	2	78	31	YPL031C	
XVI	-	377577	378389	813	419	2	86	48	YPL090C	
XVI	-	5818	5988	171	40	2	59	43	YPL283C	
XVI	+	795030	795377	348	17	2	59	58	YPR132W	
XVI	+	218647	218726	80	20	2	40	86	YPL175W	
XII	-	766187	766249	63	5	2	49	55	YLR316C	
XII	-	548653	548763	111	25	1	37	35	YLR199C	
XII	-	548599	548763	165	79	1	64	37	YLR199C	
XII	+	1067085	1067476	392	173	1	56	37	YLR464W	
VII	-	787800	788178	379	13	1	76	22	YGR148C	
VII	-	497891	497999	109	47	1	68	33	YGR001C	
VII	-	365510	365985	476	17	1	43	26	YGL076C	
XII	+	987142	987636	495	424	1	45	51	YLR426W	
XII	+	987142	987339	198	127	1	32	38	YLR426W	
VII	+	727039	727647	609	290	1	67	27	YGR118W	
VII	+	543639	544128	490	407	1	64	20	YGR029W	
VII	+	543639	543788	150	67	1	68	33	YGR029W	
VII	+	543639	543755	117	34	1	47	32	YGR029W	
IX	-	4515	4986	472	173	1	37	56	YIL177C	
XI	+	437838	437925	88	20	1	56	16	YKL002W	
XI	+	437838	437913	76	8	1	56	40	YKL002W	
XI	+	437838	437898	61	7	1	63	35	YKL002W	
IV	-	1319746	1319816	71	25	1	66	30	YDR424C	
IV	-	1073339	1073401	63	26	1	44	56	YDR305C	
IV	-	715192	715358	167	56	1	54	43	YDR129C	
IV	-	457716	458097	382	302	1	42	58	YDR005C	
IV	-	306948	307765	818	386	1	69	28	YDL083C	
IV	-	254949	255044	96	26	1	57	43	YDL115C	
IV	+	1401801	1402144	344	40	1	46	51	YDR471W	
IV	+	652781	653504	724	19	1	24	77	YDR099W	
IV	+	122078	122222	145	46	1	53	48	YDL189W	
XIV	-	62371	62923	553	2	1	50	22	YNL302C	
XII	+	242322	242712	391	32	1	45	56	YLR048W	
XIV	+	622945	623297	353	11	1	28	73	YNL004W	
V	-	348093	348276	184	109	1	73	27	YER093C-A	
V	-	4210	4601	392	173	1	37	56	YEL076C-A	
X	+	91095	91614	520	203	1	22	75	YJL177W	
XIII	-	666942	667017	76	8	1	38	63	YMR201C	
XIII	-	666915	667017	103	19	1	61	21	YMR201C	
XIII	-	666909	667017	109	25	1	34	54	YMR201C	
XIII	-	163553	163716	164	244	1	65	27	YML056C	
XIII	+	424998	425160	163	7	1	24	75	YMR079W	
XIII	+	211445	212043	599	473	1	51	50	YML034W	
VIII	-	148110	148666	557	7	1	36	42	YHR021C	
VIII	+	251156	251226	71	22	1	53	46	YHR076W	
VIII	+	126552	127332	781	220	1	20	76	YHR010W	
II	-	60190	60697	508	4	1	59	37	YBL087C	
II	-	60150	60697	548	44	1	65	36	YBL087C	
II	+	462430	462521	92	22	1	80	16	YBR111W-A	
II	+	170677	170871	195	67	1	69	27	YBL026W	
XV	-	240935	241025	91	13	1	43	49	YOL048C	
XV	-	240512	241025	514	436	1	60	41	YOL048C	
XV	+	867150	867539	390	47	1	74	19	YOR293W	
XV	+	505938	506252	315	86	1	72	27	YOR096W	
XVI	-	729321	729481	161	65	1	76	25	YPR098C	
XVI	+	795030	795422	393	28	1	58	24	YPR132W	
XVI	+	412257	413012	756	17	1	46	46	YPL075W	
XVI	+	218647	218721	75	25	1	60	26	YPL175W	
XVI	+	173666	174107	442	35	1	51	48	YPL198W	

Novel Introns spanning a stop codon

Chromosome	Strand	Start	End	Length		Read support	Anchor – Slack 1	Anchor – Slack 2	Gene	
II	-	443706	443833	128		200	97	99	YBR101C	K
XIII	+	559783	560157	375		162	97	97	YMR147W	J
VII	+	436313	436374	62		53	97	87	YGL033W	
XVI	+	75982	76219	238		4	44	33	YPL250W-A	
VII	-	489661	490504	844		2	88	29	YGL005C	
IV	-	306806	307073	268		2	67	59	YDL083C	
XII	-	1018099	1018187	89		2	55	97	YLR441C	
IV	-	1170293	1170342	50		1	54	41	YDR348C	
IV	-	8489	8930	442		1	39	61	YDL246C	
IV	+	364568	364858	291		1	68	28	YDL051W	
XIV	+	616067	616412	346		1	70	24	YNL009W	
XIV	+	604172	604316	145		1	65	31	YNL016W	
X	-	129278	129514	237		1	44	33	YJL155C	
XVI	+	932590	933026	437		1	52	40	YPR196W	
XVI	+	932586	932991	406		1	59	42	YPR196W	

Novel Introns (preserving reading frame)

Chromosome	Strand	Start	End	Length		Read support	Anchor – Slack 1	Anchor – Slack 2	Gene	
VII	-	883015	883140	126		291	98	90	YGR192C	
VII	-	253186	253248	63		66	88	96	YGL136C	H
VI	+	107341	107442	102		50	94	92	YFL014W	
XI	+	220778	220831	54		43	91	72	YKL117W	
IV	+	600933	601031	99		41	85	92	YDR077W	
VIII	-	33627	33698	72		25	91	68	YHL034C	
VII	-	437563	437628	66		23	90	92	YGL031C	
VI	-	220902	221108	207		13	95	91	YFR031C-A	

Novel Introns (disrupting reading frame)

Chromosome	Strand	Start	End	Length		Read support	Anchor – Slack 1	Anchor – Slack 2	Gene	
VII	+	439382	439479	98		56	58	98	YGL030W	
VII	+	439382	439475	94		17	56	98	YGL030W	

XII	+	1073669	1073966	298		41	91	90	YLR467W
XV	+	1086634	1086931	298		36	91	90	YOR396W
IV	+	1527482	1527779	298		35	91	90	YDR545W

5' alternative splice site

Chromosome	Strand	Start	End	Length	length difference t	Read support	Anchor – Slack 1	Anchor – Slack 2	Gene
VI	-	223441	223727	287	44	1275	99	41	YFR032C-A
VII	-	534474	534781	308	4	665	97	84	YGR027C
XIII	+	211441	211570	130	4	127	95	95	YML034W
X	+	74114	74204	91	317	76	62	97	YJL191W
XII	+	856989	857057	69	414	28	86	92	YLR367W
II	+	170621	170804	184	56	25	51	87	YBL026W
VI	-	64600	64856	257	64	16	82	93	YFL034C-A
VII	+	439098	439323	226	4	13	48	96	YGL030W
VII	-	365527	365969	443	16	7	94	37	YGL076C
XVI	+	412262	412995	734	5	7	68	93	YPL075W
XV	-	92441	92521	81	309	6	68	61	YOL121C
IX	+	317047	317171	125	275	5	98	70	YIL018W
XII	+	856570	857057	488	5	5	100	54	YLR367W
XII	+	263277	263594	318	71	5	84	74	YLR061W
XVI	+	218663	218746	84	16	5	39	86	YPL175W
IX	+	317018	317171	154	246	4	73	28	YIL018W
VIII	+	126548	127112	565	4	4	33	69	YHR010W
XI	-	618375	618526	152	216	3	63	72	YKR094C
XIV	+	331453	331837	385	127	3	88	94	YNL162W
X	+	91052	91411	360	43	3	44	84	YJL177W
XVI	+	795135	795394	260	105	3	59	92	YPR132W
XII	-	712726	713247	522	92	3	97	24	YLR287C-A
VII	+	556128	556307	180	297	2	33	95	YGR034W
IX	-	4688	4959	272	27	2	98	31	YIL177C
IV	-	239399	239533	135	24	2	63	34	YDL125C
IV	+	337494	337634	141	31	2	23	86	YDL064W
IV	+	308523	308792	270	95	2	34	89	YDL082W
XIV	+	557575	557684	110	36	2	42	80	YNL038W
V	+	307656	307848	193	91	2	25	74	YER074W-A
VIII	-	148117	148508	392	158	2	61	57	YHR021C
II	+	333321	333386	66	445	2	51	84	YBR048W
II	+	168597	168808	212	172	2	53	94	YBL027W
XVI	-	345445	345714	270	118	2	85	51	YPL109C
XVI	+	794891	795394	504	139	2	32	44	YPR132W
XVI	+	412889	412995	107	632	2	75	34	YPL075W
XVI	+	406726	407067	342	79	2	45	52	YPL079W
VII	-	497938	498032	95	33	1	19	75	YGR001C
VII	+	1084775	1085093	319	108	1	26	74	YGR296W
VII	+	311229	311526	298	213	1	68	33	YGL103W
IX	-	256555	256828	274	198	1	61	17	YIL052C
XI	-	93367	93515	149	50	1	42	59	YKL186C
IV	-	340811	341155	345	64	1	18	53	YDL061C
IV	+	653138	653523	386	357	1	77	22	YDR099W
XIV	-	534875	534980	106	15	1	85	15	YNL050C
XIV	-	494525	494632	108	341	1	56	27	YNL069C
XIV	-	5869	6187	319	108	1	71	27	YNL339C
V	-	348202	348286	85	10	1	9	91	YER093C-A
X	-	469184	469405	222	142	1	61	27	YJR021C
XIII	-	666934	667111	178	94	1	23	47	YMR201C
XIII	-	140091	140188	98	5	1	40	31	YML067C
III	-	101634	101790	157	90	1	36	65	YCL012C
VIII	-	148117	148821	705	155	1	62	30	YHR021C
VIII	-	148117	148401	285	265	1	24	74	YHR021C
II	+	168555	168808	254	130	1	23	50	YBL027W
XVI	-	5778	6096	319	108	1	71	27	YPL283C
XVI	+	795166	795394	229	136	1	49	50	YPR132W
XVI	+	406955	407067	113	308	1	52	49	YPL079W
XII	-	712726	712939	214	216	1	41	54	YLR287C-A
