# Supplementary material for: Alternative Splicing in Next Generation Sequencing Data of Saccharomyces cerevisiae
Source: PLoS One. 2015 Oct 15;10(10):e0140487. doi: 10.1371/journal.pone.0140487 (PMC4607428; doi:10.1371/journal.pone.0140487)
Supplement: S1 Fig — PCR gels of 10 tested novel introns and alternative splicing events. The expected position of PCR products confirming novel events are marked with arrows. Numbers describe the adjacent PCR product size (in bases) of the two marker bands. Chromosomal locations of the events are listed below. The S. cerevisiae strain BY4741 (MATa; his3 Δ1; leu2 Δ0; met15Δ0; ura3Δ0) (Euroscarf, Frankfurt, Germany) was cultivated in YPD at 30°C. Exponentially growing S. cerevisiae cells at an OD595 of 0.8 were collected and used for preparation of RNA applying the SV Total RNA Isolation System (Promega, Madison, USA). For first strand cDNA preparation 1 μg of total RNA was transcribed in a 25 μl reaction using the M-MLV reverse transcriptase (Promega, Madison, USA) according to manufacturer instructions. 1 μl of the resulting first strand cDNA was used as template in 50 μl PCR reactions (0.2 mM dNTPs; 1 μM primer; 1.5 mM MgCl2; 35 cycles using an annealing temperature of 55°C and 1min synthesis time at 72°C) applying splice variant specific primer pairs (S2 Table) and GoTaq G2 DNA Polymerase (Promega, Madison, USA). 10 μl of the PCR reactions were analyzed on 2% agarose gels stained with DNA Stain Clear G (Serva, Heidelberg, Germany). The above experiment was repeated three times. The first time without any sequencing attempt. The second time sequencing failed. The third time sub-clonig and subsequent sequencing was done: For sub-cloning, 50 µl PCR reactions were completely separated on 2% agarose gels and the bands corresponding to the splice product were purified using the MiniElute PCR Purification Kit (Qiagen, Hilden, Germany). The purified splice product was sub-cloned using the TOPO TA Cloning Kit for Sequencing (Life Technologies, Carlsbad, USA) according to the manufacturer’s protocol. Positive clones were selected after DNA-preparation by control PCRs with the respective primer set and further analyzed by sequencing (MWG Eurofins, Ebersberg, Germany). In Experiment J the “expec [file pone.0140487.s002.pdf]

PCR gels of 10 tested novel introns and alternative splicing events. The expected position of PCR products confirming novel events are marked with arrows. Numbers describe the adjacent PCR product size of the two marker bands in bases.

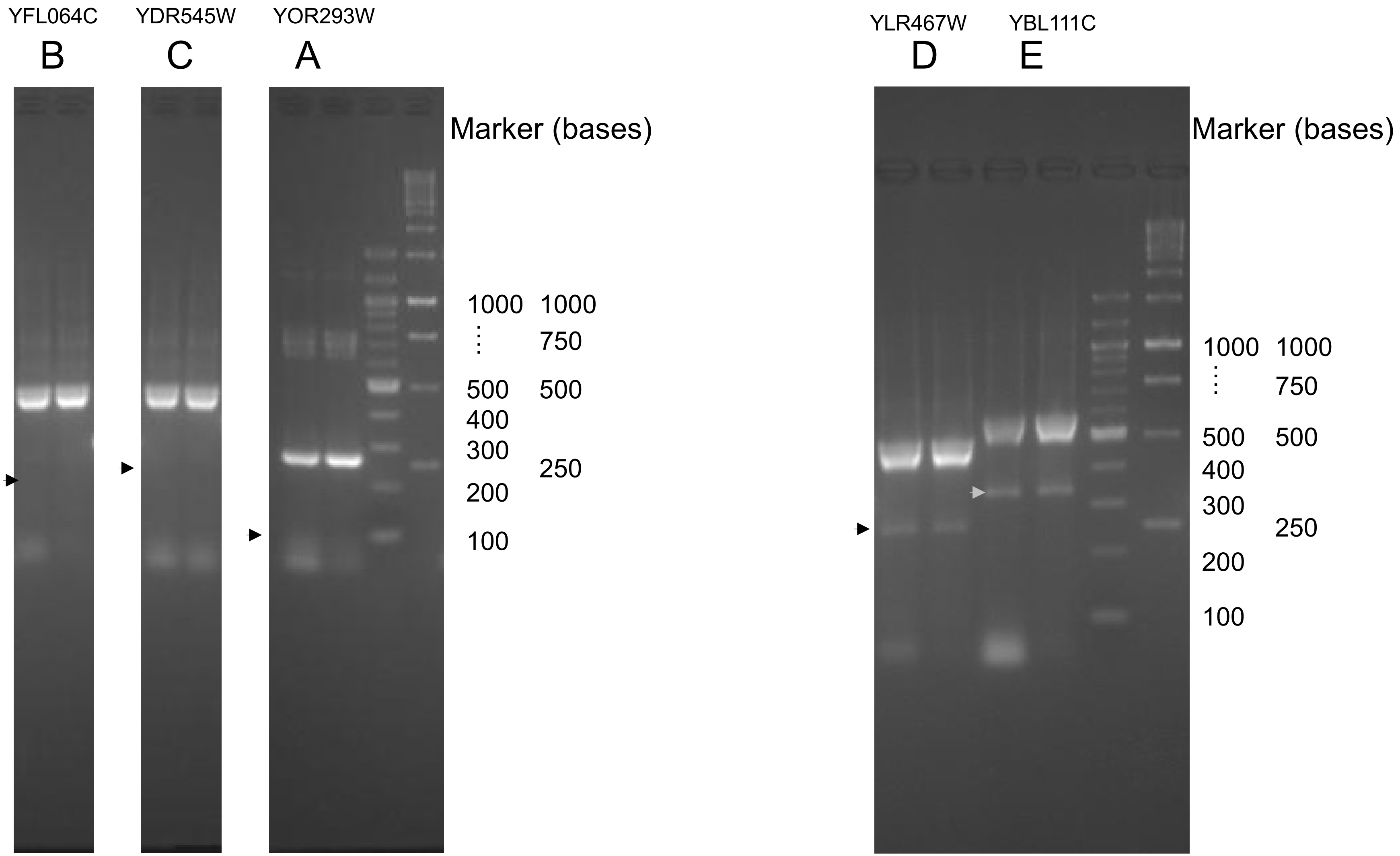

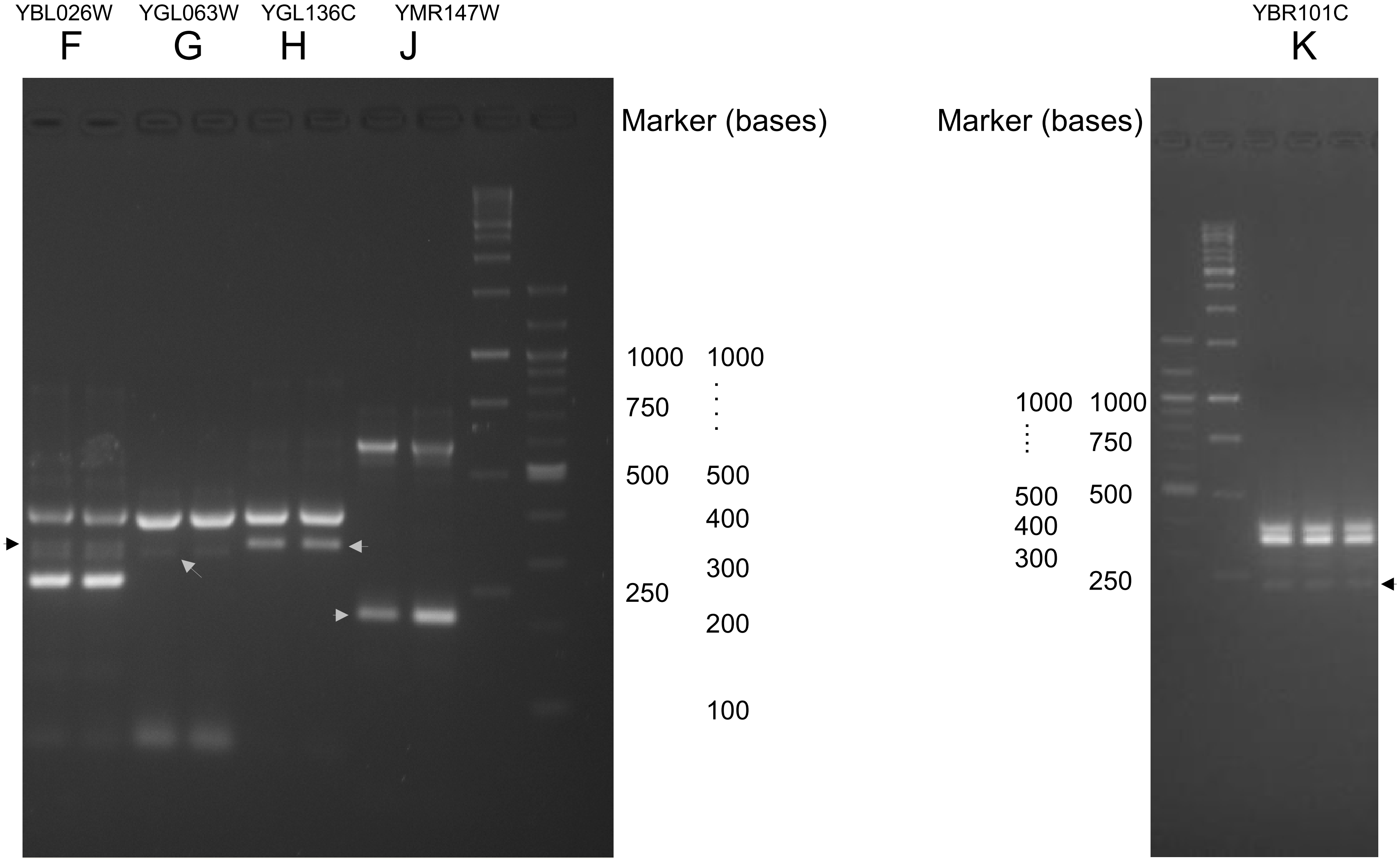
YBL026W
F
YGL063W
G
YGL136C
H
YMR147W
J
Marker (bases)
1000
750
500
250
1000
500
400
300
200
100
YBR101C
K
Marker (bases)
1000
500
400
300
1000
750
500
250

name	chr	start	end	expected fragment length	expected novel fragment length (marked with arrow at gel pictures)
A (YOR293W)	XV	867150	867746	265, 703	107
B (YFL064C)	VI	3957	4168	430	219
C (YDR545W)	IV	1526005	1526211	445	239
D (YLR467W)	XII	1072192	1072398	458	252
E (YBL111C)	II	5120	5336	551	335
F (YBL026W)	II	170677	170758	388, 259	307
G (YGL063W)	VII	383486	383566	388	308
H (YGL136C)	VII	253186	253249	387	324
J (YMR147W)	XIII	559783	560158	585	210
K (YBR101C)	II	443706	443834	351	223

Successful sequencing of “expected novel fragment length” J (zoom in):

```
PRIMER p18: XIII 559783,560158 strand:
p18:1

primer: ATTTTCAATCCTTCGTTGAAAAATG--------------------------------------------------------------------------------------------------------------------------------------------------------------GCATTATTATTAGGATTTCCCTATCAA
      : |||||||||||||||||||||||||                                                                                                                                                              |||||||||||||||||||||||||||
 clone: ATTTTCAATCCTTCGTTGAAAAATGCAAACAGGTTTTCTACAACTTCAGTCTGCAAACAGTAGAAAAATTAAATGCTCTACAAAATAGCTTGTACGAAGTATTTTGGATTATTTTTATTTATTTAAACTACTGGTTCCCCAATGTGGGTGACTATGTGAGCAATACTTTTGGACAACAAGATAGCATTATTATTAGGATTTCCCTATCAA

genome: ATTTTCAATCCTTCGTTGAAAAATGCAAACAGGTTTTCTACAACTTCAGTCTGCAAACAGTAGAAAAATTAAATGCTCTACAAAATAGCTTGTACGAAGTATTTTGGATTATTTTTATTTATTTAAACTACTGGTTCCCCAATGTGGGTGACTATGTGAGGTATGTCTGTCGTAGCTTCTCGCGCCATAACGAAATTGCCCAATTACTAACACGTATTTTTACTTACAATATCAACCACCTCCACTAATGAACAGGCACAGTATAGAAAAAAAATCAGATCTGCATTTAAAAGGCTGTCTTTTCCAATGATCCTCGACCTTAGCGCGGCTAATCAATTCTACTCACTTTTTTGTTCGCTTTTTTTTTTTTTGACCCGTTGAACCAAGCGGGTTAATTATTTTTTAAGGATAAAGGCTACCACAATCATGAGAGAGACACCATGTACTTGCAGGATTCTGATATTTTTTCTATGCTTGTTTGCTGACATGCAGATTCGGAGCTGGTTTTGATATTTCATAATCACCTATTTTTGAAGCAATACTTTTGGACAACAAGATAGCATTATTATTAGGATTTCCCTATCAA
      : ||||||||||||||||||||||||||||||||||||||||||||||||||||||||||||||||||||||||||||||||||||||||||||||||||||||||||||||||||||||||||||||||||||||||||||||||||||||||||||||||                                                                                                                                                                                                                                                                                        ||||||||||||||||||||||||||||||||||||||||||||||||||
 clone: ATTTTCAATCCTTCGTTGAAAAATGCAAACAGGTTTTCTACAACTTCAGTCTGCAAACAGTAGAAAAATTAAATGCTCTACAAAATAGCTTGTACGAAGTATTTTGGATTATTTTTATTTATTTAAACTACTGGTTCCCCAATGTGGGTGACTATGTGAG----------------------------------------------------------------------------------------------------------------------------------------------------------------------------------------------------------------------------------------------------------------------------------------------------------------------------------------------------------------------------------------CAATACTTTTGGACAACAAGATAGCATTATTATTAGGATTTCCCTATCAA
 check: --------------------------------------------------------------------------------------------------------------------------------------------AATGTGGGTGACTATGTGAG----------------------------------------------------------------------------------------------------------------------------------------------------------------------------------------------------------------------------------------------------------------------------------------------------------------------------------------------------------------------------CAATACTTTTGGACAACAAG------------------------------
```

The Primer pair J (`primer`) resulted in the cloned sequence (`clone`).

Aligned to the genome (`genome`) the sequence matches the expected splicing event (`check`).
