# Supplementary material for: Alternative Splicing in Next Generation Sequencing Data of Saccharomyces cerevisiae
Source: PLoS One. 2015 Oct 15;10(10):e0140487. doi: 10.1371/journal.pone.0140487 (PMC4607428; doi:10.1371/journal.pone.0140487)
Supplement: S2 Table — Location and sequences of the PCR primers used. (PDF) [file pone.0140487.s003.pdf]

name	chr	start	end	expected fragment length	expected novel fragment lengtl	primer1	primer2
A	XV	867150	867746	265, 703	107	ACAAGATCCACCAATACTTATTCCA	TGGGTTTCTTTCTTGAATGTAGGTA
B	VI	3957	4168	430	219	GAAGTATCCAAGAGAATATCCACGA	GCCTATCGGAAACTTTCATTTTATT
C	IV	1526005	1526211	445	239	AAGTATCCAAGAGAATATCCACGAA	GCCTATCGGAAACTTTCATTTTATT
D	XII	1072192	1072398	458	252	GGTTCTCCTCGACTAAGCAGATAGT	CCAAGTCGTTTTTGTTATTTAGAGC
E	II	5120	5336	551	335	TTAAGATACTGTGCACCATGGAAAT	ACCAGTTCTGAATTTTACCACTTTG
F	II	170677	170758	388, 259	307	TAACACCCTAGCAAGTATGCTTTTC	GCAAATTCGTATCTACCATGTTCTT
G	VII	383486	383566	388	308	AAGACAAATATTTACAGGCCAGAGA	CGTGTACACCCTTGTCTGTTCTT
H	VII	253186	253249	387	324	TTTTCACCTCTGATGACATTTATGA	AATAGGTCTCCTTTGATTCGTCTCT
J	XIII	559783	560158	585	210	ATTTTCAATCCTTCGTTGAAAAATG	TTGATAGGGAAATCCTAATAATAATGC
K	II	443706	443834	351	223	ATTGAAAGTACGATTGAGTGCTTGT	TTTTAATGAATTCCATTTTACAGAAAG
