# Supplementary material for: Alternative Splicing in Next Generation Sequencing Data of Saccharomyces cerevisiae
Source: PLoS One. 2015 Oct 15;10(10):e0140487. doi: 10.1371/journal.pone.0140487 (PMC4607428; doi:10.1371/journal.pone.0140487)
Supplement: S2 Fig — We compare our results with the recently published results of Kawashima et al. Venn diagrams compare potential intron assignments at two different levels of confidence in our analysis to introns reported by Kawashima et al. In each plot the set “All” contains all introns fulfilling our length requirements and presenting a valid splice signal. The set “Only accepted” only contains introns that additionally fulfill our support and junction quality criteria. A) Overlap limited to annotated introns. We observe a large overlap of about 90% introns reported in Kawashima et al. [4] and about 2/3 of our introns. B) Overlap without annotated introns. In these diagrams we compare true alternative splicing events reported here and by Kawashima et al. The overlap among proposed isoforms is not expected to be high, since our analysis pipeline is different (e.g. Kawashima et al. also report alternative splice sites without canonical splice signals) and AS is likely to be context specific. Some isoforms are exactly found in both studies, so those results are reproducible under different experimental and technical conditions. c) Out of the highly confident AS events reported in the tables in the main text, five are also reported by Kawashima et al. Strikingly, all of them are at the 3’ splice site, and four preserve the reading frame. The set “Reported” only contains the predicted introns that are listed in the tables in this manuscript excluding any novel introns (n = 20), because those are not considered by Kawashima et al. The potential introns in the sets “All” and “Only accepted” are derived following our workflow, depicted in Fig 1 in the manuscript. Summing up “All” (107 + 180 + 804 + 56) results in 1,147 potential introns and summing up “Only accepted” (103 + 174 + 157 + 13) results in 447 predicted introns. (These numbers also appear in Figs 2(A) and 3) (PDF) [file pone.0140487.s004.pdf]

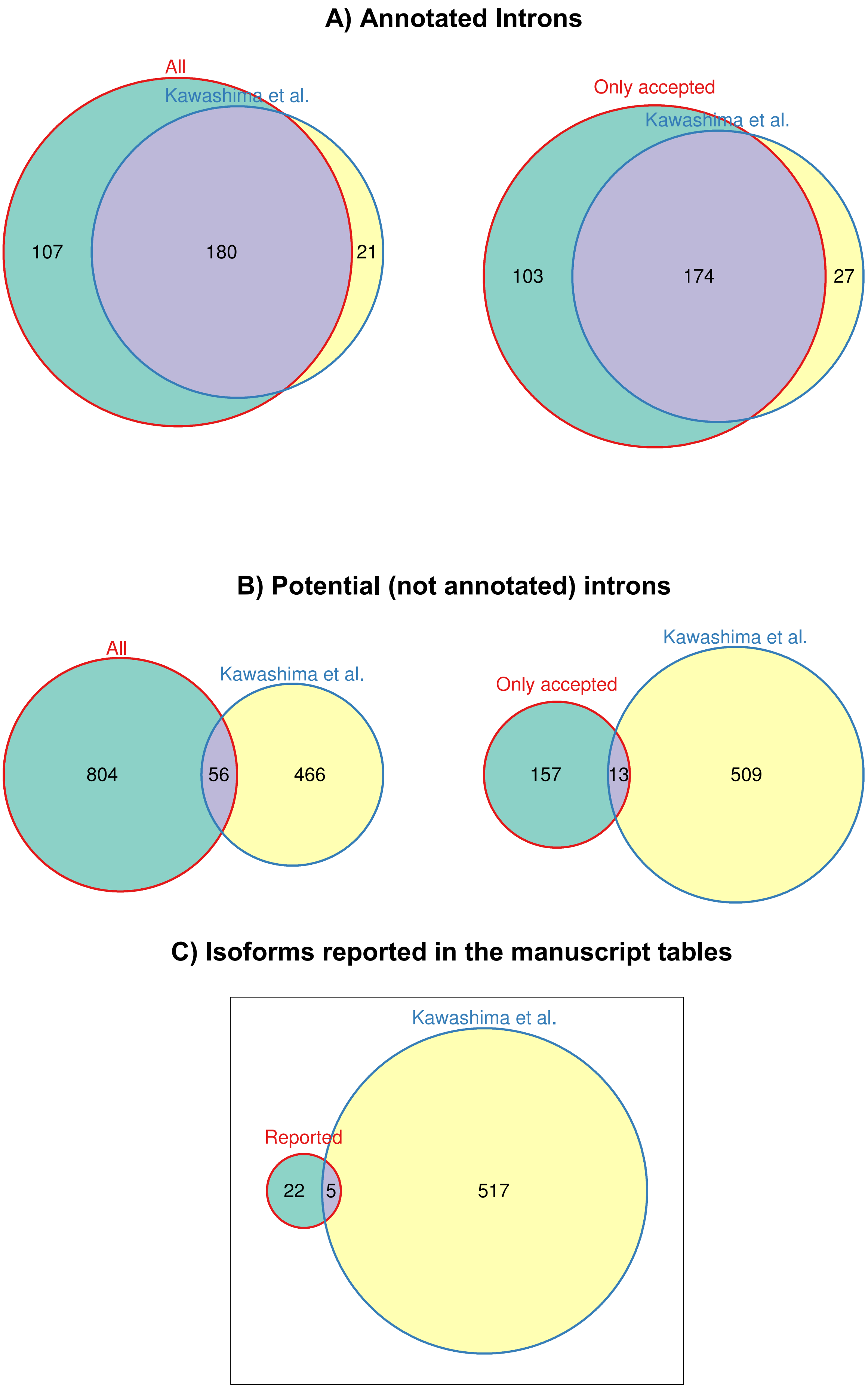
A) Annotated Introns
All
Kawashima et al.
107
180
21
Only accepted
Kawashima et al.
103
174
27
B) Potential (not annotated) introns
All
Kawashima et al.
804
56
466
Only accepted
Kawashima et al.
157
13
509
C) Isoforms reported in the manuscript tables
Kawashima et al.
Reported
22
5
517

The five Isoforms reported in both, this study and Kawashima et al.:

(3' alternative splice site, preserving the reading frame:)

Chromosome	Strand	Fom	To	Length difference	Read support	Gene
XI	+	155272	155636	-18	86	YKL157W
II	+	393181	393507	-3	28	YBR078W
XII	+	1067085	1067303	-60	22	YLR464W
II	+	653369	653524	+72	16	YBR215W

(3' alternative splice site, disrupting the reading frame:)

II	+	170677	170757	47	81	YBL026W
