# Supplementary material for: Alternative Splicing in Next Generation Sequencing Data of Saccharomyces cerevisiae
Source: PLoS One. 2015 Oct 15;10(10):e0140487. doi: 10.1371/journal.pone.0140487 (PMC4607428; doi:10.1371/journal.pone.0140487)
Supplement: S3 Fig — The data published by Kawashima et al. [4] enables us to compare the support of isoforms with high read support in wild type (WT) S. cerevisiae to NMD defective (knockout) strains. To this end, we mapped the data by ourselves and analyzed the results. In the boxplots outliers are removed, and mean values are given as additional information (red numbers). In summary, we confirm the findings of Kawashima et al. Additionally, we detect some unknown AS events that seem to be unaffected by NMD: Panel A shows how the split read support for annotated introns changes between WT and the three NMD defective strains (d1, d2, d3). Note that annotated introns have a higher median and mean read support in WT, than in any knockout strain. This changes among the unannotated introns in Panel B. Panel B shows the read support for predicted, unannotated introns. By requiring read support in at least two knockout strains, we gain confidence for each predicted intron. The negative fold change from knockout to WT is a result of efficient NMD in WT. This confirms the findings of Kawashima et al. In panel C we show all the predicted introns that are supported by at least two reads in WT, and by at least one read in one of the knockout strains. As a result, we are confident about the predicted introns in WT and able to compare them to the knockout strains. The boxplot clearly shows that the lack of NMD (in the knockout strains) has little or no effect on isoforms that are confidently expressed in WT. Consequently, such isoforms are unlikely targets of NMD in WT and, therefore, more likely functional. Also, there are other nuclear RNA decay pathways than NMD that might target those transcripts. Scatter plot D shows the same data as box plot B (including outliers): for each predicted intron, the WT read support is plotted against the mean read support of the knockout strains. In particular, the predicted introns with high support in WT do not show a significant fold change, so those transcrip [file pone.0140487.s005.pdf]

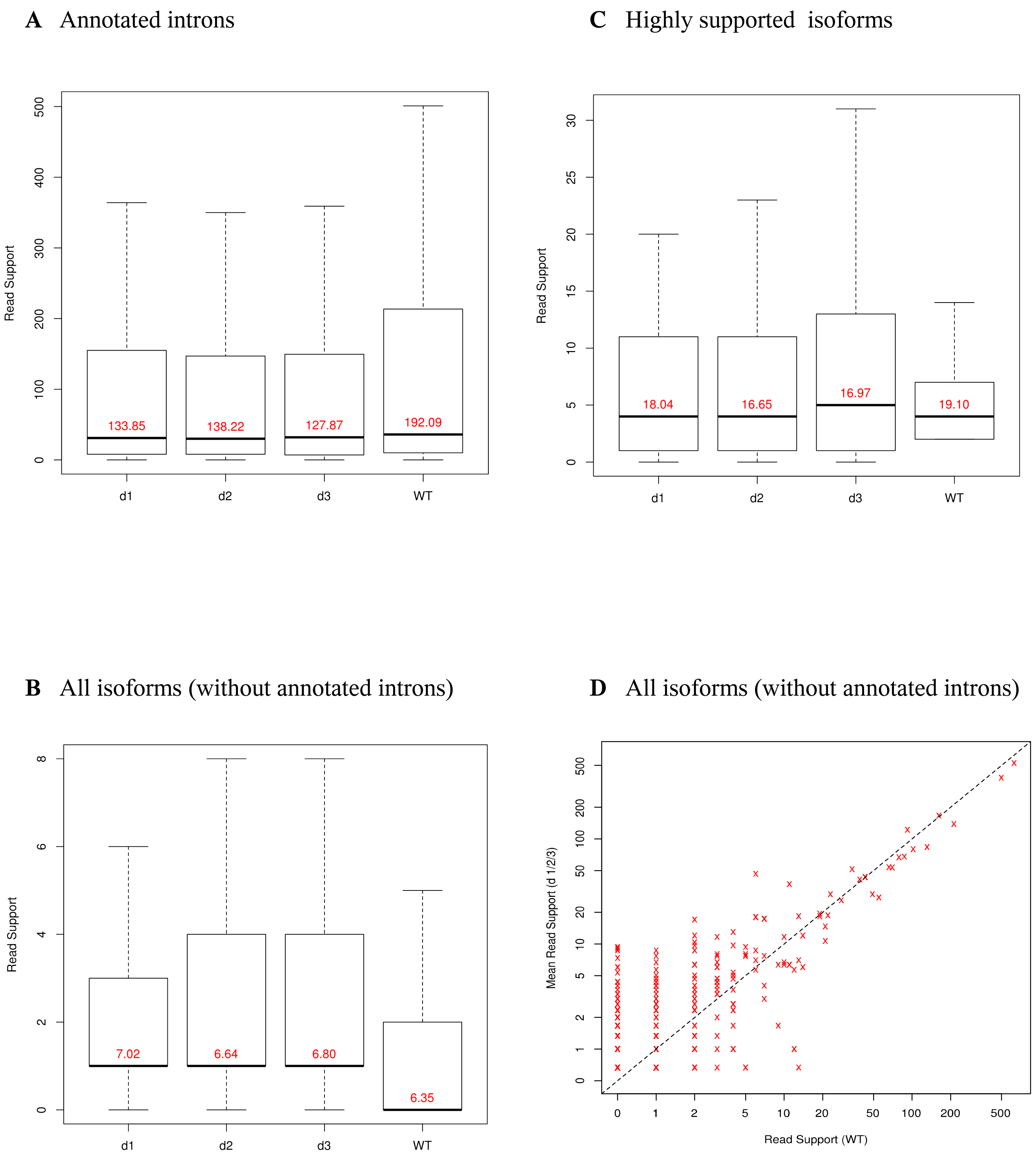
A Annotated introns
Read Support
0
100
200
300
400
500
133.85
138.22
127.87
192.09
d1
d2
d3
WT
C Highly supported isoforms
Read Support
0
5
10
15
20
25
30
18.04
16.65
16.97
19.10
d1
d2
d3
WT
B All isoforms (without annotated introns)
Read Support
0
2
4
6
8
7.02
6.64
6.80
6.35
d1
d2
d3
WT
D All isoforms (without annotated introns)
Mean Read Support (d 1/2/3)
0
1
2
5
10
20
50
100
200
500
Read Support (WT)
