# Supplementary material for: Alternative Splicing in Next Generation Sequencing Data of Saccharomyces cerevisiae
Source: PLoS One. 2015 Oct 15;10(10):e0140487. doi: 10.1371/journal.pone.0140487 (PMC4607428; doi:10.1371/journal.pone.0140487)

Read support for validated potential introns (Intron Length < 1003)

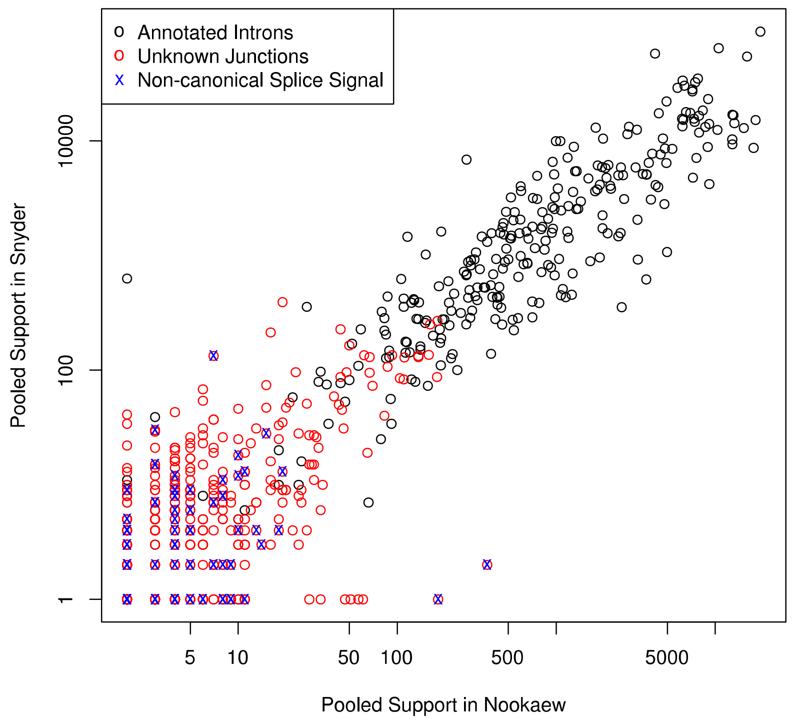

Read Support for validated potential introns (unfiltered)

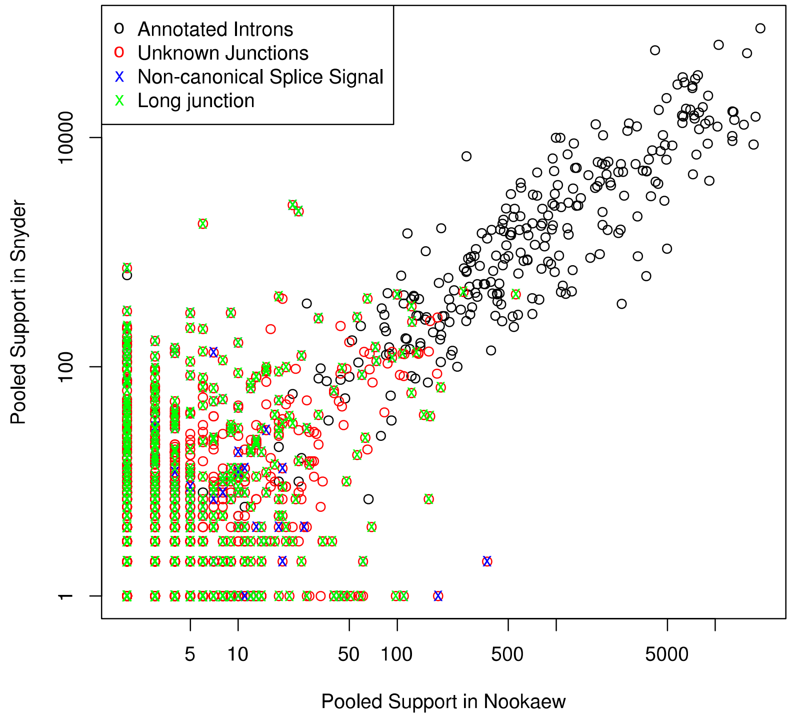

Supplement: S4 Fig — Some additional evidence for a predicted intron would be its validation in a different, independent dataset. We successfully validated the most confident intron predictions by detecting their split reads in data from Waern and Snyder [31]. In this figure we quantitatively compare the validation of potential introns by their read support in our original dataset (Nookaew et al. [25]) and the independent dataset (Waern and Snyder). Reads are pooled across experimental conditions and replicates. Figure on top: Evidently, annotated introns show high read support in either dataset and their read support seems to be correlated among datasets (Pearson’s ρ = 0.71). Among the unknown splice junctions (red circle), the correlation drops (ρ = 0.43); finally, those with a non-canonical splice signal (blue x) tend toward lower read support in general and the read support appears not to be correlated (ρ = −0.01). In the figure at the bottom we additionally show potential introns that do not pass our length filter (green x). The majority of those long introns shows a low read support and no correlation among datasets (ρ = 0.16), qualifying them as possible read or mapping errors. However, there are some potential introns covering long stretches of the S. cerevisiae genome. Some of these can also be validated in the independent dataset (green crosses around the center of the plot). These read mappings can either be explained by genomic rearrangements in the used strains or point toward post-transcriptional mechanisms in S. cerevisiae. Since there is no known mechanism explaining such transgenic splicings, the presence of these transcriptional products needs to be validated by other means. (PDF) [file pone.0140487.s006.pdf]
