# Supplementary figures and images for: Alternative Splicing in Next Generation Sequencing Data of Saccharomyces cerevisiae
Source: PLoS One. 2015 Oct 15;10(10):e0140487. doi: 10.1371/journal.pone.0140487 (PMC4607428; doi:10.1371/journal.pone.0140487)

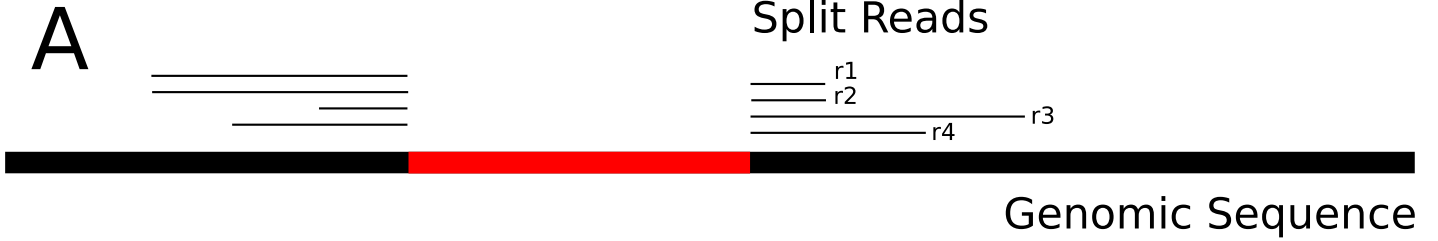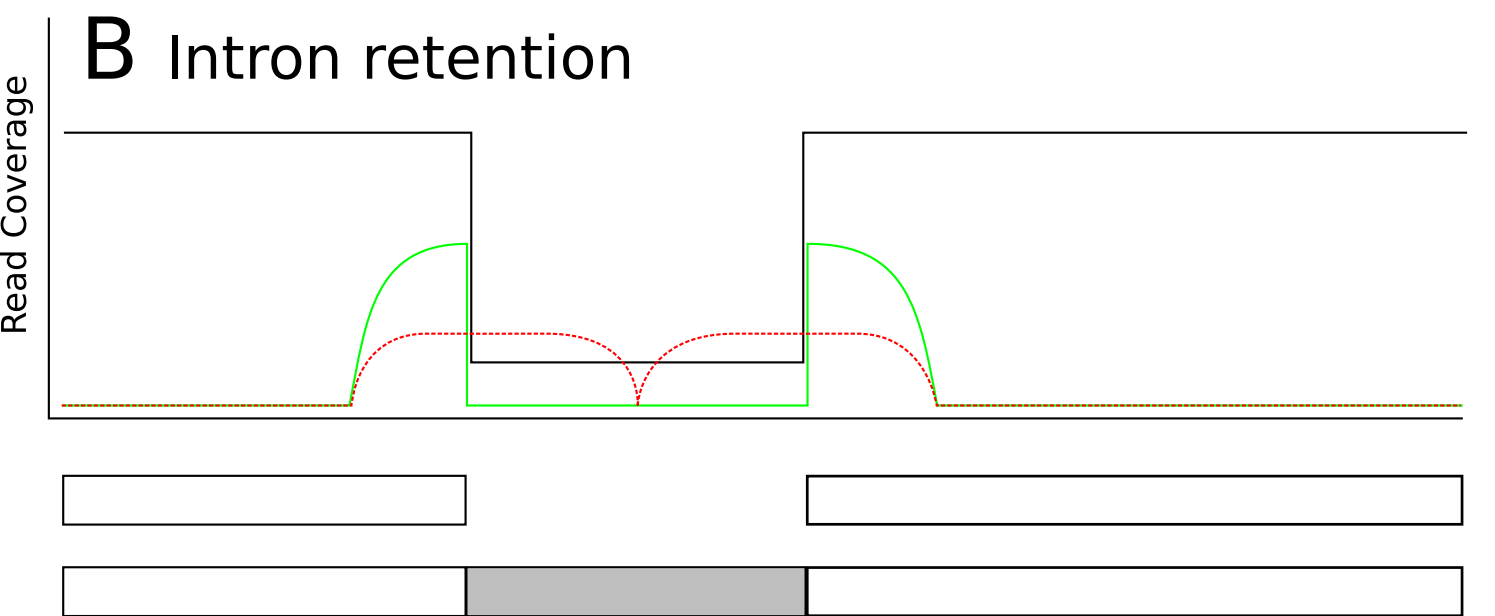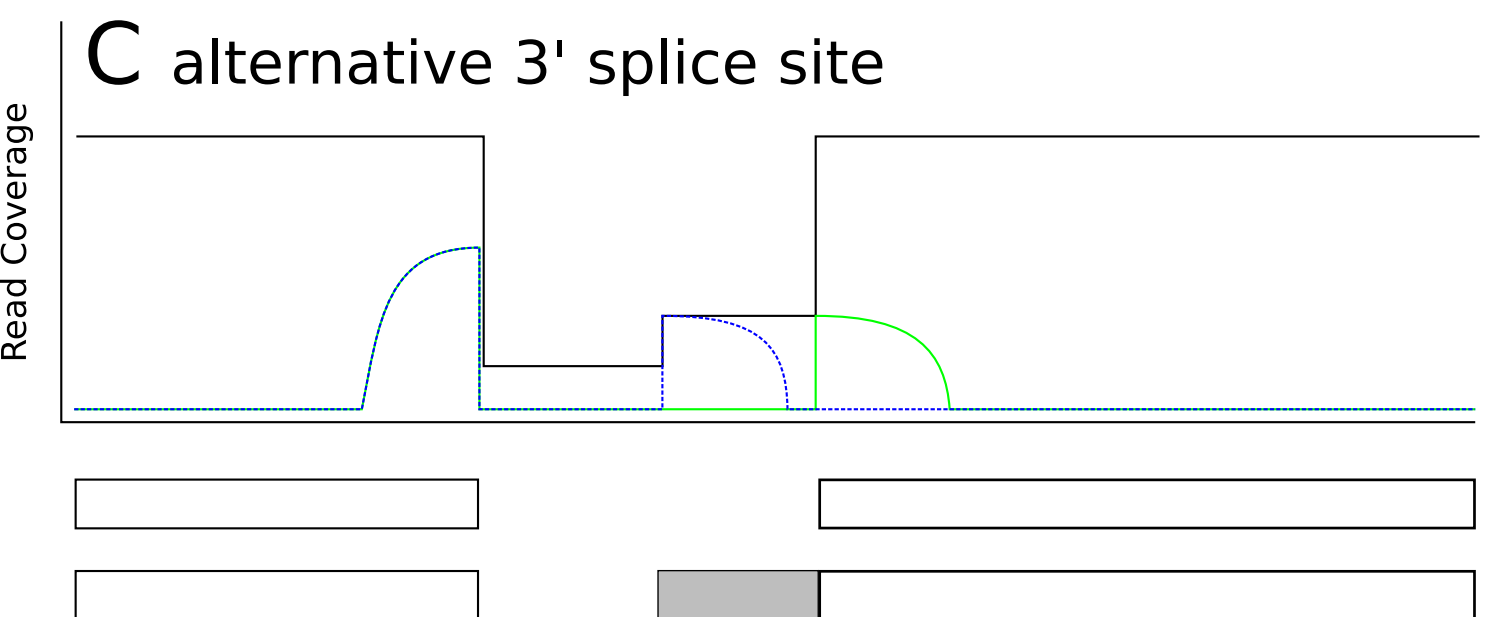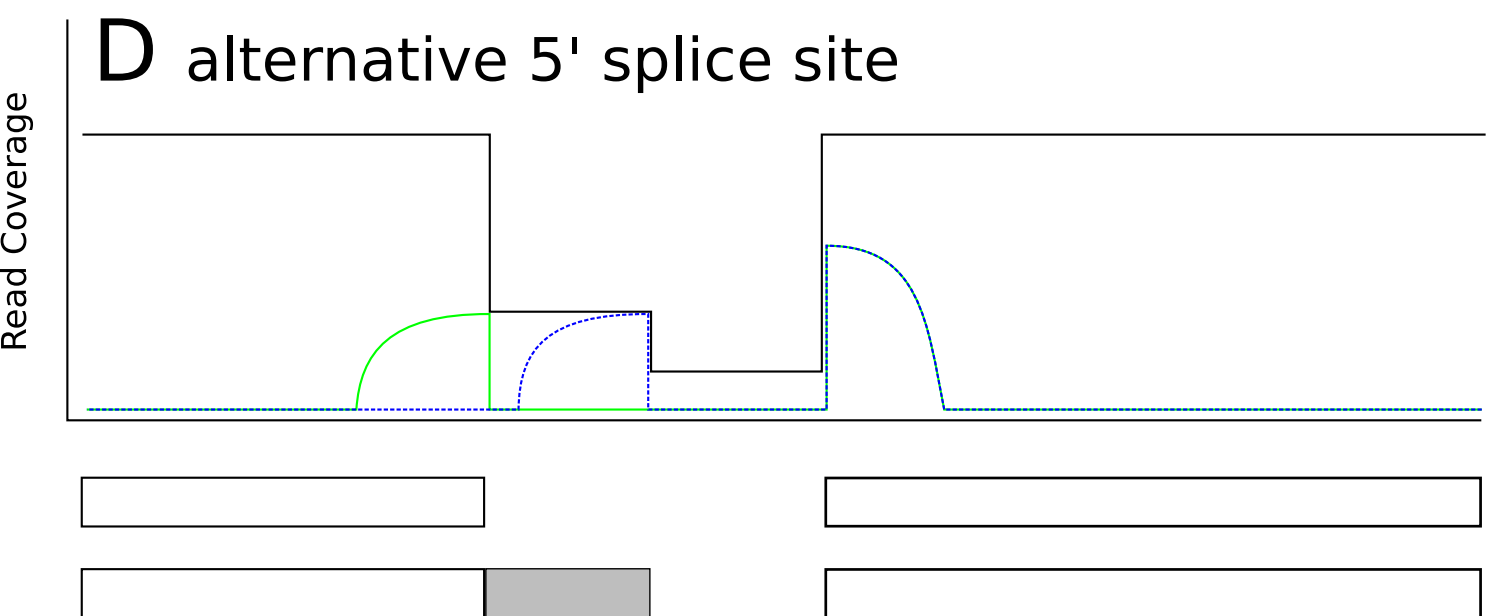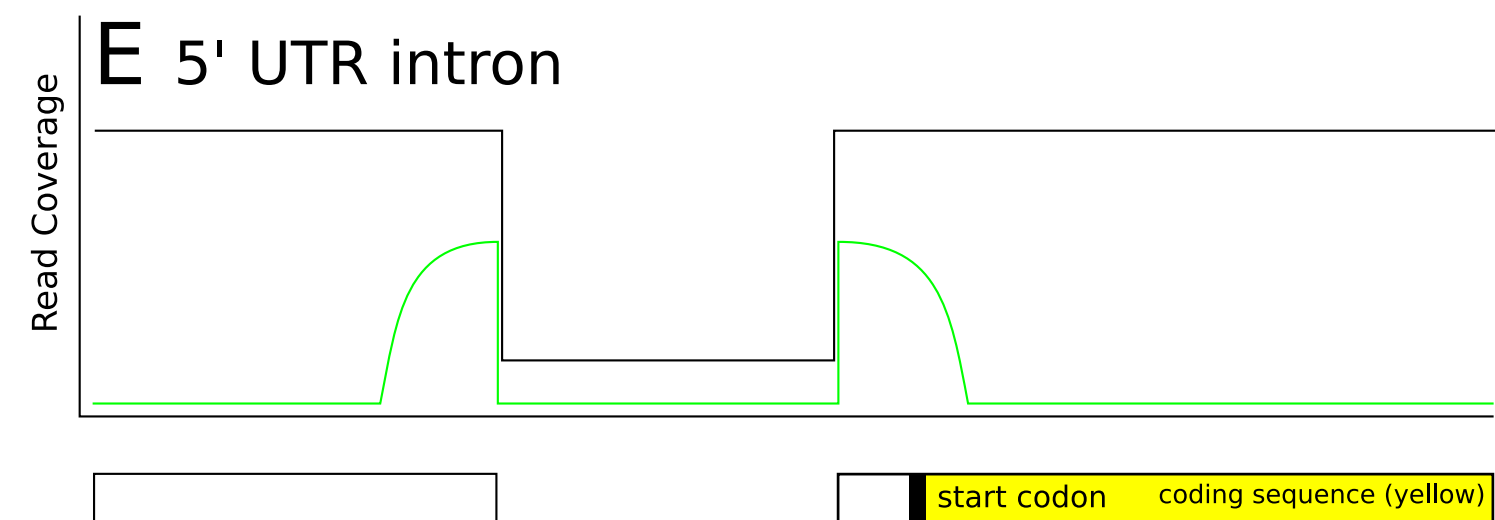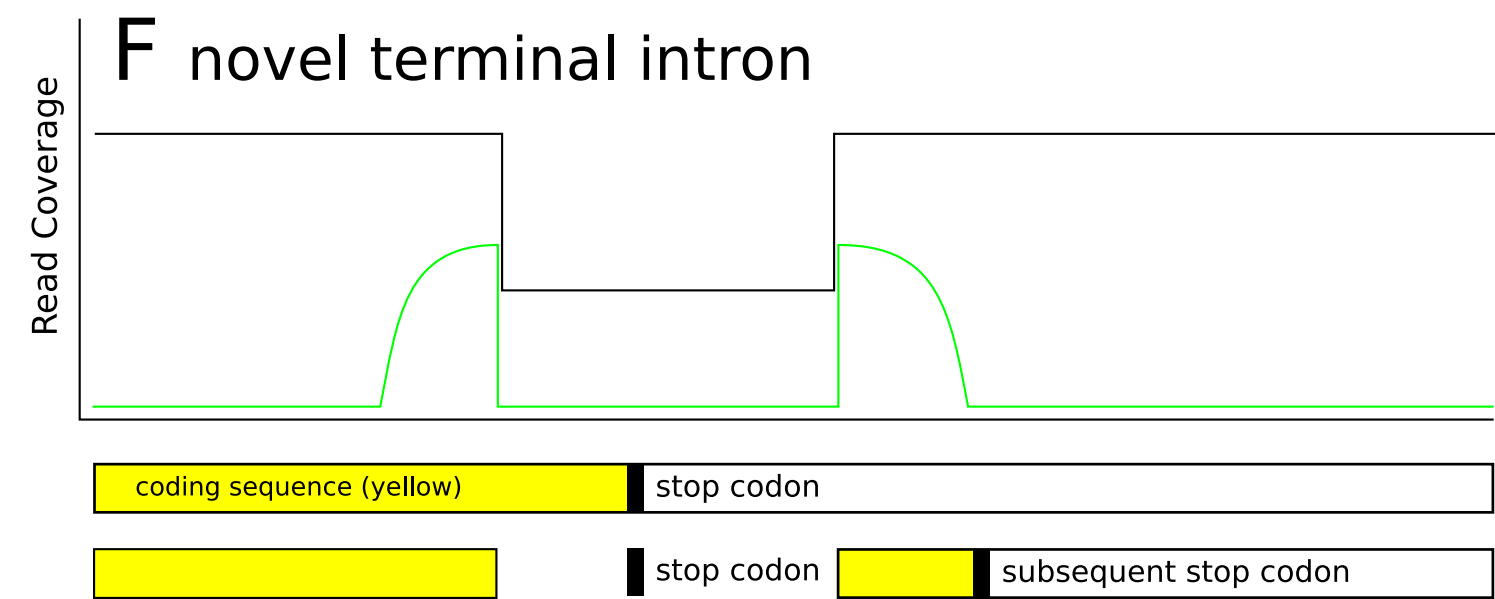

Supplement: S5 Fig — Alternative transcript models together with their idealized read coverage in next generation sequencing data. The black line represents the total read coverage of all mapped reads. The green, red and blue lines represent characteristic reads for certain isoforms. (A) Split reads defining an intron. Reads r1 through r4 are mapped to the genome in a spliced form; the potential intron is supported by 4 reads. Reads r1 and r2 represent the same fragment, because they have exactly the same sequence and hence the same start and end position on the genome. As a result, the potential intron is supported by three distinct fragments. (B) Intron retention. The total read coverage (black) drops in the area of the intron and the intronic area is spanned by split reads (green). Ungapped (red) reads containing the 3’ or 5’ splice site are evidence of intron retention. (C) Alternative 3’ splice site. The total read coverage is lower in the intron, but shows an increase towards the 3’ site. There are two different types of overlapping split reads (green and blue), using the same 5’ splice site, but different 3’ splice sites. (D) Alternative 5’ splice site. Similar to alternative 3’ splice sites, but mirrored. (E) 5’ UTR intron. The intron is located before the start codon and the coding sequence (yellow) starts downstream. (F) Novel terminal intron. The intron overlaps the annotated stop codon and splices it out, extending the coding sequence to the next downstream stop codon. (PDF) [file pone.0140487.s008.pdf]
